# Anthocyanins From Sweet Potatoes (*Ipomoea batatas*): Bioavailability, Mechanisms of Action, and Therapeutic Potential in Diabetes and Metabolic Disorders

**DOI:** 10.1002/fsn3.70895

**Published:** 2025-09-04

**Authors:** Sammra Maqsood, Nosiba S. Basher, Muhammad Tayyab Arshad, Ali Ikram, Douglas S. Kalman, Md. Sakhawot Hossain, Emmanuel Laryea, Nasir A. Ibrahim

**Affiliations:** ^1^ National Institute of Food Science and Technology, University of Agriculture Faisalabad Faisalabad Pakistan; ^2^ Department of Biology, College of Sciences Imam Mohammad Ibn Saud Islamic University (IMSIU) Riyadh Saudi Arabia; ^3^ Functional Food and Nutrition Program, Faculty of Agro‐Industry Prince of Songkla University Hatyai Songkhla Thailand; ^4^ University Institute of Food Science and Technology The University of Lahore Lahore Pakistan; ^5^ Dr. Kiran C. Patel College of Osteopathic Medicine Nova Southeastern University Fort Lauderdale Florida USA; ^6^ Department of Nutrition and Food Technology Jashore University of Science and Technology Jashore Bangladesh; ^7^ Department of Food Science and Technology Kwame Nkrumah University of Science and Technology Kumasi Ghana

**Keywords:** diabetes risk reduction, functional food, metabolic health, sweet potato

## Abstract

*Ipomoea batatas* , commonly known as sweet potato, is an increasingly valued functional food because of its vivid coloration and rich bioactive compounds, especially anthocyanins and carotenoids, such as ipomoeaxanthin. This review focuses on the bioavailability, mechanisms of action, and therapeutic potential of sweet potato‐derived anthocyanins in diabetes and metabolic disorders. Anthocyanins, which are plant pigments, exhibit high antioxidant activity by scavenging free radicals and stimulating endogenous antioxidant enzymes such as catalase and superoxide dismutase, thereby protecting cellular structures from damage and reducing oxidative damage in vital metabolic organs such as the pancreas, liver, brain, and muscles. Anthocyanins also increase insulin sensitivity, regulate glucose metabolism, and regulate enzymes involved in carbohydrate digestion, thus reducing the risk of diabetes. In addition, anthocyanins inhibit low‐grade chronic inflammation by inhibiting the inflammatory mediators TNF‐α, IL‐6, and NF‐κB signaling pathways implicated in the progression of type 2 diabetes. Clinical evidence supports preclinical animal models and ongoing human trials favoring sweet potato consumption to enhance glucose control and decrease insulin resistance. However, challenges remain regarding the poor bioavailability of anthocyanins and the need for stronger human studies. In addition to anthocyanins, sweet potatoes contain diverse nutrients that contribute to their metabolic health. This review highlights the significance of sweet potatoes as a functional food ingredient in diabetes prevention diets and encourages new processing methods that can sustain their bioactive potential.

## Graphical abstract

This review highlights the nutrient diversity in this variety of sweet potatoes beyond anthocyanins, sweet potatoes' place in diabetes risk reduction diets for diabetes, and the importance of creative methods of processing that keep them bioactive. The author highlighted the increased recognition of sweet potatoes as a functional food that heralds the potential of sweet potatoes as part of an overall healthy diet to be utilized in metabolic health interventions and disease risk reduction.

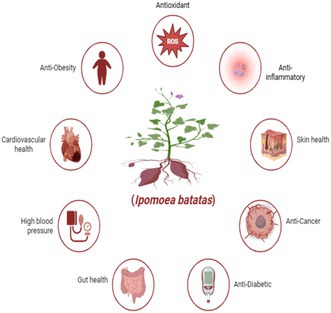

## 1 Introduction

Sweet potatoes ( *Ipomoea batatas* ) are among the most versatile and nutrient‐rich root crops. They are staple in the world. Their prominence in food security and sustainable agriculture is underscored by their adaptability to wide climatic environments ranging from tropical to subtropical and temperate locations (Cartabiano‐Leite et al. 2020; Campos et al. 2017). In low‐income regions, sweet potatoes are highly valued for their flexibility in culinary applications and their essential role in reducing malnutrition and providing crucial nutrients (Alam 2021). The cultural status of this crop in traditional and modern food systems is exemplified by the diverse ways in which sweet potatoes are prepared, such as boiling, baking, frying, or processing into flour, starch, and even drinks (Alam 2021).

Sweet potato is one of the most nutritious crops grown in tropical and subtropical regions, and is known as *Ipomoea batatas* (L.) Lam. The crop is a heterozygous hexaploid of the Convolvulaceae family (Zhao et al. 2013; Sapakhova et al. 2023). It is the most widely planted food crop in the world, with China at the top of the list, producing more than 50% of the world's yield (Zhao et al. 2013; Sapakhova et al. 2023). Sweet potato is an important crop for food security, animal feed, and raw materials in the industry owing to its low input requirements, high water‐use efficiency, and poor soil tolerance (Zhao et al. 2013; Sapakhova et al. 2023). Nevertheless, biotic and abiotic challenges imperil its production, which requires investigation on disease‐resistant varieties and improvement of crop resistance through molecular breeding (Zhao et al. 2013; Sapakhova et al. 2023). From an agronomic perspective, sweet potatoes are useful because they require minimal inputs (financial, physical, etc.) and are tolerant to drought and poor soil, thus becoming a reliable food supply during challenging circumstances (Lebot 2010). Apart from agronomic advantages, the roots contain high amounts of dietary fiber, carbohydrates, vitamins A (pro‐vitamin A), C, and B6, and essential minerals, such as potassium and manganese (Bovell‐Benjamin 2007).

Sweet potatoes are an important part of the human diet, especially in regions where food insecurity remains critical (Escobar‐Puentes et al. 2022). Purple‐fleshed sweet potatoes have garnered special attention among other varieties of sweet potatoes because of their high anthocyanin content. Total carbohydrates present up to 72.10 g/100 g (dry weight, DW), free sugars (reducing sugars): 1.01–5.94 g/100 g (varies by variety and maturity stage), maltose: 11.98% of fresh weight (FW), sucrose: 8.33% of FW, glucose + fructose: 6.52% of FW, starch: 56.7 g/100 g DW, amylose: 18.2%–27.2%, rapidly digestible starch (RDS): 40.66%–53.50%, slowly digestible starch (SDS): 10.40%–23.84%, resistant starch (RS): 29.25%–43.50% dietary fiber, total dietary fiber: ~16% DW, insoluble fiber: higher than soluble fiber, peels: > 60% dietary fiber (77.6% insoluble fiber), protein content: 2.33 g/100 g DW (flesh), amino acids (highest content): glutamic acid: 565.75 mg/kg, aspartate: 479.74 mg/kg, arginine: 413.54 mg/kg, alanine: 371.87 mg/kg, leucine: 336.67 mg/kg, leaves: 16.2 to 30.3 g/100 g DW (higher protein content than flesh) while moisture content: 62.6%–73.6% (Rosell et al. 2024). Bioactive compounds, such as anthocyanins, including cyanidin 3‐sophoroside‐5‐glucoside, cyanidin 3‐(6,6′‐dicaffeoyl‐sophoroside)‐5‐glucoside, cyanidin 3‐(6,6′‐caffeoyl‐p‐hydroxybenzoyl sophoroside)‐5‐glucoside, cyanidin 3‐(6,6′‐caffeoylferuloylsophoroside)‐5‐glucoside, peonidin 3‐(6,6′‐dicaffeoyl‐sophoroside)‐5‐glucoside, and peonidin 3‐(6,6′‐caffeoyl‐p‐hydroxybenzoyl sophoroside)‐5‐glucoside are present in purple sweet potato (Rosell et al. 2024).

Other bioactive compounds found in sweet potatoes are flavonoids, non‐flavonoids, carotenoids, and organic acids, which provide 85 kcal per 100 g FW (edible portion), and polysaccharides may contain proteins and uric acids, enhancing the antioxidant activity in purple sweet potato (Rosell et al. 2024). The deep purple color of these potatoes is attributed to anthocyanins, a family of water‐soluble flavonoids known for their antioxidant properties (Jiang et al. 2022; Sugata et al. 2015). These bioactive compounds are vital for scavenging free radicals, reducing oxidative stress, and reducing the risk of developing chronic diseases such as diabetes, heart disease, and cancer (Vishnu et al. 2019). There is considerable variation in the anthocyanin content of sweet potatoes owing to genetic and environmental influences such as soil quality, climate, and production methods. An investigation of functional foods and nutraceuticals has focused on purple‐fleshed sweet potatoes because they contain a much higher anthocyanin level than other types (Wang et al. 2018). Their popularity within the food industry, particularly in health‐conscious products such as snack foods, smoothies, and supplements, also derives from their bright color and health benefits (Islam 2024).

According to recent research, anthocyanin‐rich sweet potatoes possess anti‐diabetic effects associated with enhanced glucose metabolism, improved insulin sensitivity, and reduced inflammation (Escobar‐Puentes et al. 2022; Sugata et al. 2015). Moreover, their antioxidative properties are associated with enhanced cellular health, which is vital for curing metabolic and age‐related syndromes (Alam 2021). In addition to their health benefits, anthocyanin‐rich sweet potatoes are a great resource to combat food insecurity. Sweet potatoes are increasingly being added to nutrition enhancement programs in disadvantaged regions, particularly developing countries. Sweet potatoes have been considered a “superfood” for the next century because of their ability to provide bioactive compounds and combat malnutrition (Islam 2024). Sweet potatoes have a rich cultural history in several countries. For example, orange‐fleshed varieties have been utilized in Africa as a remedy for vitamin A insufficiency (Campos et al. 2017).

Similarly, purple‐fleshed sweet potatoes are heavily consumed in food, drinks, and desserts across Asia and the US, signifying their integration into various food cultures (Jiang et al. 2022). Sweet potatoes are important in local economies, where these products represent a source of revenue for smallholder farmers and have been crucial in agro‐industrial systems, further intensifying their cultural and financial significance (Llamera and Llamera 2024). Sweet potatoes, particularly those rich in anthocyanins, flavonoids, non‐flavonoids, carotenoids, and organic acids, are exclusive hubs of cultural, agricultural, and nutritional value. These tubers remain a critical topic in discussions on worldwide health, sustainable agriculture, and food security as research continues to discover more applications and health benefits. Sweet potatoes further characterize the flavonoid class of pigments originating from plants, of which examples comprise such species: anthocyanins in vivid red, purple, and blue colors. Water solubility occurs in fruits, vegetables, and other crops such as sweet potatoes ( *Ipomoea batatas* ) (Bovell‐Benjamin 2007).

Bioactive compounds are vital for attracting pollinators, protecting them from UV light, and bestowing resistance to infection (Ellong et al. 2014). Anthocyanins are important for human health because of their anti‐inflammatory and antioxidant properties. Anthocyanins act as free radical scavengers, reducing oxidative stress and subsequent chronic diseases such as diabetes, cancer, and cardiovascular diseases (Ji et al. 2015). In addition to their antioxidant properties, bioactive compounds, such as anthocyanins, flavonoids, and carotenoids, modulate inflammatory processes and are associated with enhanced immune function (El Far and Taie 2009). Environmental factors such as pH, temperature, and anthocyanin chemical configuration and constancy determine the bioactivity (Escobar‐Puentes et al. 2022). The anthocyanins from purple‐fleshed sweet potatoes have a more sophisticated antioxidant capability than those from other sources, especially those based on cyanidin and peonidin (Teow et al. 2007; Truong et al. 2018). The culmination of this research has led to the development and promotion of this variety of sweet potatoes as a functional food for health and as a source of nutrients and phytonutrients that are also used in dietary ingredients and supplements.

Sweet potatoes containing high amounts of anthocyanins, flavonoids, and flavonols, such as kaempferol, quercetin, myricetin, and fisetin, have been consumed by humans, especially in regions of Asia, with traditional diets often rich in cultivars bearing deep purple flesh (Lebot 2010; Baky et al. 2022). Originally desired for their colorful hue and flavorful taste, varieties of the crop gained appeal when health benefits were found (Sugata et al. 2015). Anthocyanins are gaining interest because of the recent increase in attention paid to efficient foods. As stated by Hue et al., functional meals are meals that provide additional health benefits besides essential nourishment, typically for specific diseases such as inflammation, oxidative stress, or metabolic diseases (Hue et al. 2012). Purple sweet potatoes are an example of these products owing to their high anthocyanin levels. Another reason for the popularity of anthocyanins is the increasing consumer demand for plants and other naturally derived bioactive compounds (Vishnu et al. 2019). Their use in various applications such as baked products, dietary supplements, and beverages demonstrates their business potential (Wang et al. 2018; Kourouma et al. 2020).

In addition, advancements in metabolomics and analytical techniques have expanded our knowledge regarding gratification and the role of anthocyanins in numerous sweet potato cultivars (Zhang et al. 2018). In conclusion, anthocyanins are the secret to the appeal and health benefits of sweet potatoes, as they are both natural pigments and bioactive compounds (Teow et al. 2007). Their historical use, exceptional quality, current research, and consumer interests demonstrate their significance in the development of human health.

This review highlights the potential of sweet potatoes as a functional food for health promotion and maintenance while noting the existence of research gaps that may limit or impact the promotion of sweet potatoes as a food for health.

## 2 Anthocyanin Types in Sweet Potatoes

**FIGURE 1:**
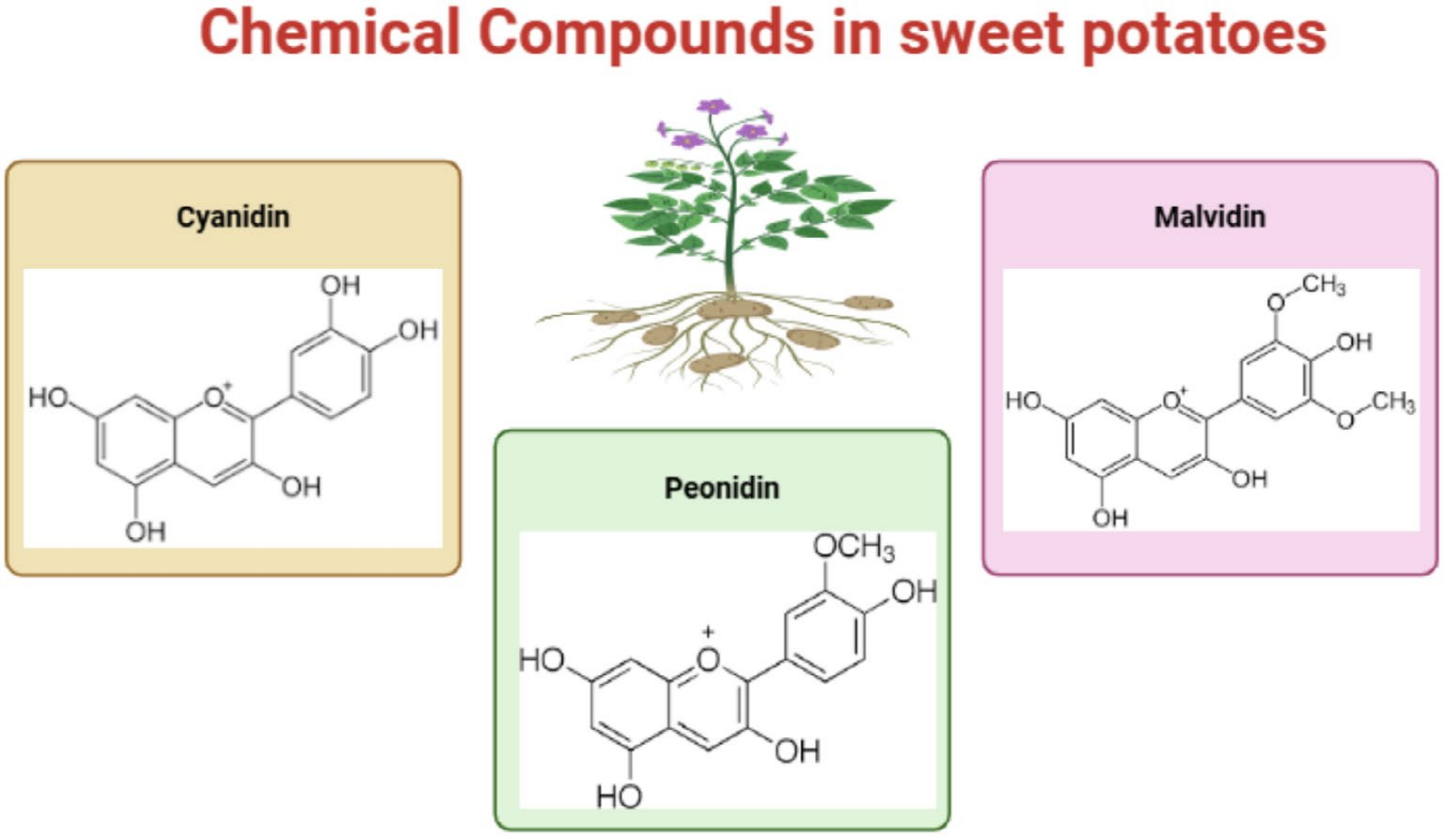
Structures of chemical compound in sweet potatoes.

Sweet potatoes, especially those with purple flesh, contain anthocyanins, which are water‐soluble pigments responsible for the bright color of the plants. As shown in Figure 1, structural variations between anthocyanins determine their bioactivity and processing stability, which are critical for their health‐promoting effects.

In addition to contributing to color, numerous pigments have significant biological properties that enhance human health. The three major anthocyanins present in sweet potatoes are glycosylated anthocyanidins: cyanidin, peonidin, and malvidin. Each of these is discussed in detail below, focusing on its structure, function, and association with sweet potatoes. Table 1 depicts the anthocyanin and proximate composition of different sweet potato varieties.

**TABLE 1:** Anthocyanin and proximate composition of different sweet potato varieties.

Sweet potato type	Data category	Parameter	Value(s)	References
Purple‐fleshed (P40)	Anthocyanins	Cyanidin 3‐sophoroside‐5‐glucoside	[M + H]^+^: 773 m/z (Fragments: 661, 449, 287)	(Su et al. 2019)
		Peonidin 3‐sophoroside‐5‐glucoside	[M + H]^+^: 787 m/z (Fragments: 595, 433, 271)	(Su et al. 2019)
		p‐hydroxybenzoylated derivatives	[M + H]^+^: 893–907 m/z	(Su et al. 2019)
		Caffeoylated derivatives	[M + H]^+^: 935–1125 m/z	(Su et al. 2019)
Orange‐fleshed	Proximate composition	Moisture (%)	62.2–78.2 (fresh)	(Neela and Fanta 2019)
		Ash (%)	0.85–4.94	(Neela and Fanta 2019)
		Protein (%)	0.58–5.83	(Neela and Fanta 2019)
		Fats (%)	0.03–1.70	(Neela and Fanta 2019)
		Starch (%)	26.34–65.41	(Neela and Fanta 2019)
		Crude fiber (%)	0.35–4.52	(Neela and Fanta 2019)
		Total carbohydrates (%)	18.3–90.17	(Neela and Fanta 2019)

### 2.1 Cyanidin

Cyanidin is the most abundant anthocyanin in sweet potatoes. It is characterized by strong antioxidant properties and a dark‐red or purple color that depends on pH. The contents of cyanidin‐3‐glucoside and cyanidin‐3‐sophoroside in purple sweet potato were made possible by glycosylating cyanidin with a sugar moiety, either glucose or sophorose. These compounds improve plant resistance to environmental and oxidative stress (Truong et al. 2010; Xu et al. 2015).

Cyanidin is known to enhance the antioxidant content of sweet potatoes because it exhibits potent free radical scavenging, prevents lipid peroxidation, and protects DNA from oxidative damage (Kano et al. 2005). Studies using sweet potato extracts have shown that, in addition to its antioxidant activity, cyanidin has anti‐inflammatory activity through the modification of inflammatory mediators, such as NF‐κB and cytokines. These properties may help to treat and prevent chronic diseases, including diabetes and cardiovascular diseases (Huang et al. 2021).

### 2.2 Peonidin

Another significant anthocyanin in sweet potatoes is peonidin, which is characterized by its reddish‐purple color and structural similarity to cyanidin but with an additional methoxy group on the B‐ring (Huang et al. 2021). This minor difference makes it a significant pigment in handled sweet potato foodstuffs because of its increased stability at various pH levels (Li et al. 2019; Montilla et al. 2010).

The primary component of sweet potatoes is peonidin‐3‐glucoside, which produces a vibrant color and bioactive characteristics. The anti‐inflammatory and anti‐diabetic properties of peonidin are noteworthy. Peonidin has been shown to improve insulin sensitivity and decrease the indicators of oxidative stress in animal models, providing a potential therapeutic application (Jiao et al. 2012). In addition, extracts from sweet potatoes with high peonidin levels were found to have prebiotic activity that affected gut microbiota composition and improved gut health (Zhang et al. 2016).

### 2.3 Malvidin

The two methoxy groups on the B‐ring of malvidin (a type of flavonoid), one of the less common but important anthocyanins in sweet potatoes, endow it with a rich violet color and higher stability than other anthocyanins (Kim et al. 2015; Khoo et al. 2017). Using advanced chromatographic methods, malvidin glycosides such as malvidin‐3‐glucoside have been detected in purple sweet potatoes (Truong et al. 2010; Lee et al. 2013). Malvidin is an essential constituent of sweet potatoes owing to its robust antioxidant and anticarcinogenic properties (Baky et al. 2022). Research has demonstrated that it can attenuate oxidative stress and inflammation by scavenging reactive oxygen species (ROS) and strengthening cellular antioxidant defenses (Xu et al. 2015).

Moreover, malvidin has shown potential in modulating glucose metabolism‐related enzymatic pathways, which may be valuable in treating diabetes (Kim et al. 2015). This differentiates sweet potatoes from other anthocyanin‐rich foods, such as berries and red cabbage, with most anthocyanins unacylated and bound to organic acids such as caffeic, ferulic, and p‐coumaric acid. Acylation in food research, heat conduct, and storage improves the constancy of anthocyanins (Huang et al. 2021; Sun et al. 2018).

Pigments from sweet potatoes are resistant to degradation through acylation of anthocyanins and, therefore, are suitable for various industrial and food applications. Multiple factors, such as the attentiveness and arrangement of anthocyanins in sweet potatoes, including postharvest processing, rising conditions, and inspiring cultivars, exist. Of all the cultivars, Ayamurasaki and Murasaki have a relatively high anthocyanin content based on cyanidin derivatives (Kim et al. 2012; Kano et al. 2005). Anthocyanin retention by cooking methods such as baking and steaming depends on different actions. For instance, the level of retained anthocyanins during steaming is higher than that retained during boiling (Hong and Koh 2016). Table 2 depicts the types of anthocyanins in sweet potatoes and their characteristics.

**TABLE 2:** Types of anthocyanins in sweet potatoes and their characteristics.

Anthocyanins type	Source	Description	Comparison to other fruits/Vegetables	References
Cyanidin	Purple sweet potato	Major anthocyanin in purple‐fleshed varieties	Common in red berries, higher stability in sweet potato	(Truong et al. 2010)
Peonidin	Purple sweet potato	Contributes to purple and reddish hues	Similar to grapes but varies in acylation	(Suda et al. 2003)
Malvidin	Purple sweet potato	Enhances dark‐purple pigmentation	Found in blueberries, lower in concentration in sweet potatoes	(Montilla et al. 2010)
Delphinidin	Purple sweet potato	Provides antioxidant properties	Abundant in berries, lesser presence in sweet potato	(Ji et al. 2015)
Petunidin	Sweet potato	Minor anthocyanin contributing to coloration	Rare in vegetables, common in flowers	(Xu et al. 2015)
Acylated cyanidin	Sweet potato	Stabilized anthocyanin with extended shelf life	Specific to tuberous crops	(Kim et al. 2012)
Acylated peonidin	Sweet potato	Increased thermal stability	Less frequent in fruits compared to sweet potatoes	(Li et al. 2019)
Acylated malvidin	Sweet potato	Enhances anthocyanin structural integrity	Uncommon in other foods, unique to sweet potato	(Hu et al. 2016)
Mono‐acylated anthocyanins	Sweet potato	Single acyl group contributes to stability	Lower occurrence in berries and grapes	(Vishnu et al. 2019)
Di‐acylated anthocyanins	Purple sweet potato	Higher stability in extreme pH	Rare in most fruits and vegetables	(Huang et al. 2021)
Glycosylated cyanidin	Sweet potato	Bound with sugars for enhanced solubility	Common in fruits but different sugar types in sweet potato	(Truong et al. 2010)
Glycosylated peonidin	Sweet potato	Affects solubility and absorption	Present in berries with different glycosides	(Li et al. 2019)
Glycosylated malvidin	Sweet potato	Enhances bioavailability	Found in red wines but differs in sweet potato	(Montilla et al. 2010)
Anthocyanidin derivatives	Sweet potato	Variety of derivatives in purple‐fleshed types	Greater diversity compared to standard crops	(Bennett et al. 2021)
Precursor anthocyanins	Sweet potato	Early‐stage compounds in biosynthesis	Limited to specialized crops	(Vishnu et al. 2019)
Thermally stable anthocyanins	Sweet potato	Withstands high cooking temperatures	Rare in most crops except purple root vegetables	(Kim et al. 2015)
Enzymatically stabilized	Sweet potato	Resistant to enzymatic degradation	Different from softer fruits like strawberries	(Lim et al. 2013)
High molecular weight anthocyanins	Sweet potato	Unique polymeric anthocyanins	Rare across common dietary vegetables	(Sigurdson et al. 2019)
Anthocyanin‐rich complexes	Sweet potato	Associated with polysaccharides	Rare compared to fruits like cherries	(He et al. 2015)
Bioactive anthocyanins	Sweet potato	Enhances anti‐inflammatory effects	Higher concentration than some berries	(Kano et al. 2005)
Pigment‐stabilizing compounds	Sweet potato	Acts with co‐pigments for longevity	Differ in berries and other tubers	(Sun et al. 2018)
Diverse sugar attachments	Sweet potato	Affects flavor and stability	Unique sugars compared to fruits	(Truong et al. 2010)
Non‐acylated anthocyanins	Sweet potato	Less stable but more bioavailable	Common in cherries and strawberries	(Cai et al. 2016)
Anthocyanin complexes with iron	Sweet potato	Iron‐binding stabilizes color	Unusual compared to leafy greens	(Wang et al. 2017)
Radical‐scavenging anthocyanins	Sweet potato	Enhanced antioxidant activity	Higher compared to potatoes	(Zhang et al. 2016)
Anthocyanin esters	Sweet potato	Specific ester‐linked forms	Rare in most fruits and vegetables	(Huang et al. 2021)
Multifunctional anthocyanins	Sweet potato	Combines multiple health benefits	Concentration varies significantly across crops	(Truong et al. 2018)
Anthocyanin modulators	Sweet potato	Influences gut microbiota	Rarely observed in other vegetables	(Zhang et al. 2016)

## 3 Factors: Influencing the Concentration of Anthocyanins in Sweet Potatoes

Purple‐fleshed sweet potatoes contain high amounts of bioactive molecules termed anthocyanins, which are known for their health benefits and antioxidant properties. Concentration and bioavailability depend on various factors such as cultivar, growing conditions, and cooking methods. The anthocyanin content of the cultivars differed significantly. Anthocyanins in biosynthetic pathways are variable in purple‐fleshed categories, making them, on average, much more concentrated than other types (Mattoo et al. 2022). Greater anthocyanin content has been associated with deeper pigmentation in Ayamurasaki and Okinawan purple cultivars (Li et al. 2019).

**FIGURE 2:**
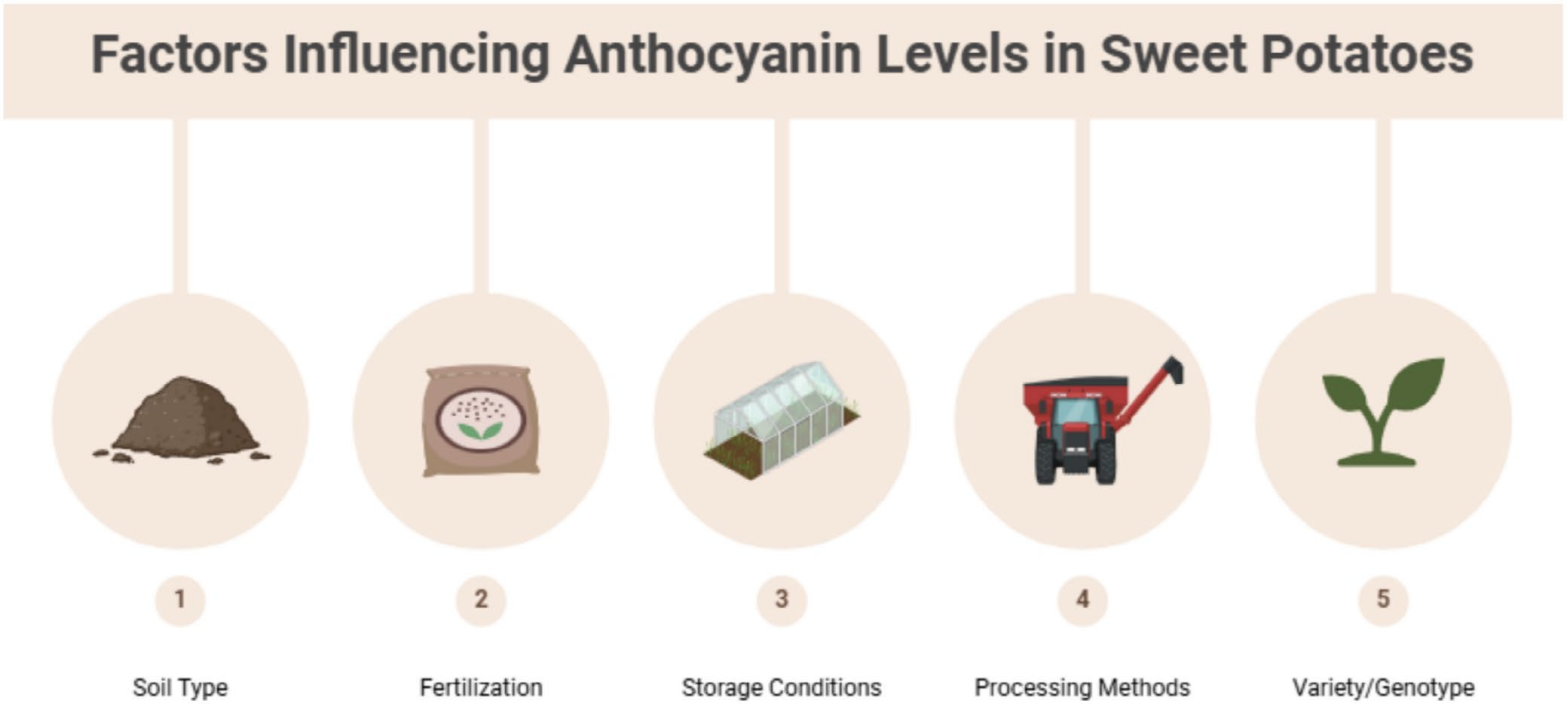
Factors influencing the anthocyanin levels in sweet potatoes.

Anthocyanin production in sweet potatoes is regulated by environmental factors including soil type, temperature, and sunlight exposure. High light intensity and cooler temperatures are positively correlated with high anthocyanin production because these conditions stimulate the expression of genes involved in anthocyanin biosynthesis (Truong et al. 2018). Conversely, to protect the plant from various stressors such as drought, the plant will also accumulate more anthocyanins as a form of protection (Villalba et al. 2024). The cooking method significantly affected the anthocyanin retention. Boiling and steaming generally retain more anthocyanins than baking or frying, which involves higher temperatures that can degrade these pigments (Carrera et al. 2021). Steaming is particularly effective because it does not subject foods to excessive heat and minimizes leaching into cooking water (Musilova et al. 2020). Because it is a faster method, microwave cooking has also been shown to retain anthocyanins (Franková et al. 2022). Figure 2 shows the factors influencing the anthocyanin levels in sweet potatoes.

The constancy of anthocyanins is dependent on storage conditions. Although extended room‐temperature storage may lead to high losses owing to oxidative processes, refrigeration can reduce the degradation of anthocyanins (Grace et al. 2014). Processing methods such as drying or freeze‐drying preserve anthocyanins much better than conventional dehydration techniques by minimizing heat and oxygen exposure (Rytel et al. 2021). Although abundant in sweet potatoes, anthocyanins have low bioavailability owing to poor absorption and high metabolism in the gastrointestinal tract (McGhie and Walton 2007).

Two factors affecting anthocyanin absorption are the conformation of the food matrix and dietary fiber content (Fang 2014a). In addition, the metabolic pathways and bioactivity of anthocyanins are determined by their structural diversity (Gui et al. 2023). Anthocyanin content and bioavailability in sweet potatoes are determined by a complex collaboration between genetic, environmental, and processing factors. Advanced processing techniques can be used to enhance the health benefits of anthocyanins by selecting appropriate cooking methods and optimizing agricultural practices.

## 4 Bioavailability of Anthocyanins in Sweet Potatoes

Many fruits and vegetables, including sweet potatoes, have deep‐red, purple, and blue colors of water‐soluble plant pigments, known as anthocyanins. Anthocyanins are flavonoids recognized for their potential health benefits and antioxidant activities. Anthocyanins have relatively poor bioavailability, indicating their ability to access the bloodstream and enter the systemic circulation (Rosell et al. 2024). Chemical composition, dietary matrix, and interaction with the gut microbiota are some of the factors that affect the absorption, metabolism, and circulation of anthocyanins (Baky et al. 2022).

Anthocyanins are absorbed by the small intestine, but this process is difficult. It is very challenging to absorb anthocyanins in their complete form because they are massive molecules and are hydrophilic. Only a fraction of the anthocyanins consumed, approximately 1% or less of the total intake, is absorbed into the blood after ingestion (McGhie and Walton 2007). Enzymes such as β‐glucosidase partially hydrolyze different types of anthocyanins in the stomach, releasing sugar moieties and enhancing their absorption (Fang 2014b). After absorption, anthocyanins undergo various phase I and II metabolic alterations, including methylation, sulfation, and glucuronidation (Gui et al. 2023). These modifications are thought to reduce the antioxidant activity of anthocyanins but enhance their solubility and stability, thereby promoting their blood circulation and renal elimination (Kay 2006). Thus, rather than being native aglycones or glycosides, anthocyanins are commonly distinguished as metabolites in the plasma. Following metabolism, anthocyanin metabolites are transported through the circulatory system to tissues such as the liver, kidneys, and brain. The biological activities of these metabolites have been shown to explain the potential anti‐inflammatory, antioxidant, and neuroprotective health benefits of anthocyanins (Fang 2014a; Gui et al. 2023).

Conversely, genetic variation, composition of the gut flora, and other dietary factors significantly influence the absorption of anthocyanins (Eker et al. 2019). Anthocyanin metabolites are excreted mainly in the urine. Although some metabolites may remain in the body for extended periods, especially if they accumulate in tissues, most of the anthocyanins are excreted within 24 h of consumption (Fang 2014a).

### 4.1 Effects of Processing on Anthocyanin Retention in Sweet Potatoes

Different food‐processing techniques affect the bioavailability and antioxidant capacity of anthocyanins in sweet potatoes. Processing alters the physical structure of sweet potatoes and affects their absorption. Examples of heat treatment include boiling, steaming, and baking, where oxidation, leaching, and degradation may cause massive losses of anthocyanins and other phytonutrients. Boiling sweet potatoes in water results in a significant loss of anthocyanin of up to 50% (Rosell et al. 2024). This is mainly because anthocyanins are water‐soluble and easily leach out during cooking. Boiling can cause high losses in anthocyanin concentrations; losses of up to 50% have been reported in some studies (Ruiz‐Rodriguez et al. 2008).

In addition, the high temperatures associated with boiling may cause chemical degradation of anthocyanins when oxygen is present. The anthocyanins in sweet potatoes were better preserved by steaming and baking than by boiling. Compared with boiling, steaming at a lower temperature and with no water contact causes less loss of anthocyanins (Palermo et al. 2014). Similarly, roasting sweet potatoes at restrained temperatures softens anthocyanins and conserves the amount of anthocyanins. Limiting exposure to elevated temperatures and oxidative conditions diminishes the deterioration of anthocyanins during cooking. High temperatures and long cooking times can chemically degrade anthocyanins through processes such as glycoside hydrolysis, which disrupts the sugar molecules committed to the anthocyanin, and oxidation, which points to the forfeiture of color and antioxidant properties (Huang et al. 2021; Oancea 2021). Therefore, sweet potatoes should be cooked for shorter durations at lower temperatures to maintain their anthocyanin content. The objective of new food‐processing technologies is to minimize the deterioration of anthocyanins that occurs during cooking. For instance, some studies have been conducted on the conservation of anthocyanins in sweet potatoes by vacuum frying, freeze‐drying, and microwave processing, which helps maintain the integrity of anthocyanins and reduce the exposure of sweet potatoes to high temperatures (Zhu et al. 2024; Barani et al. 2022). In addition, some novel methods can increase the preservation of anthocyanins, such as using natural antioxidants or steadying chemicals during processing (Nayak et al. 2015).

Anthocyanins may also be preserved through other pre‐treatment procedures such as blanching, in which sweet potatoes are temporarily submerged in hot water before cooking. For example, an investigation demonstrated that the anthocyanin quantity (88.51 mg/100 g) in purple‐fleshed sweet potato powder persisted well and improved throughout drying and storage after smearing with CEP pre‐treatment, specifically cooking and soaking in ethanol (da Silva et al. 2025). Blanching can halt the forfeiture of these beneficial materials by decelerating the degradation of anthocyanins during further cooking (Sui 2016). Generally, there is always a preference for baking and steaming techniques that require minimal water contact and result in lower temperatures, maximizing the anthocyanin content and nutritional value of sweet potatoes (Nayak et al. 2015). This is particularly important to maintain the health benefits of anthocyanin intake. Because these molecules exhibit wide metabolism but less absorption, anthocyanins exhibit relatively low bioavailability. However, their metabolites have various health benefits including anti‐inflammatory and antioxidant properties. Detailed attention to food‐processing methodologies may enhance the bioavailability and bioactivity of anthocyanins from sweet potatoes. Thermal procedures that are more tender than those applied during boiling may serve anthocyanins to preserve them better—preparation through baking and steaming. New food‐processing technologies may also improve the stability and retention of anthocyanins to maximize the health benefits of sweet potatoes.

## 5 Nutritional and Therapeutic Potential of Sweet Potatoes

**FIGURE 3:**
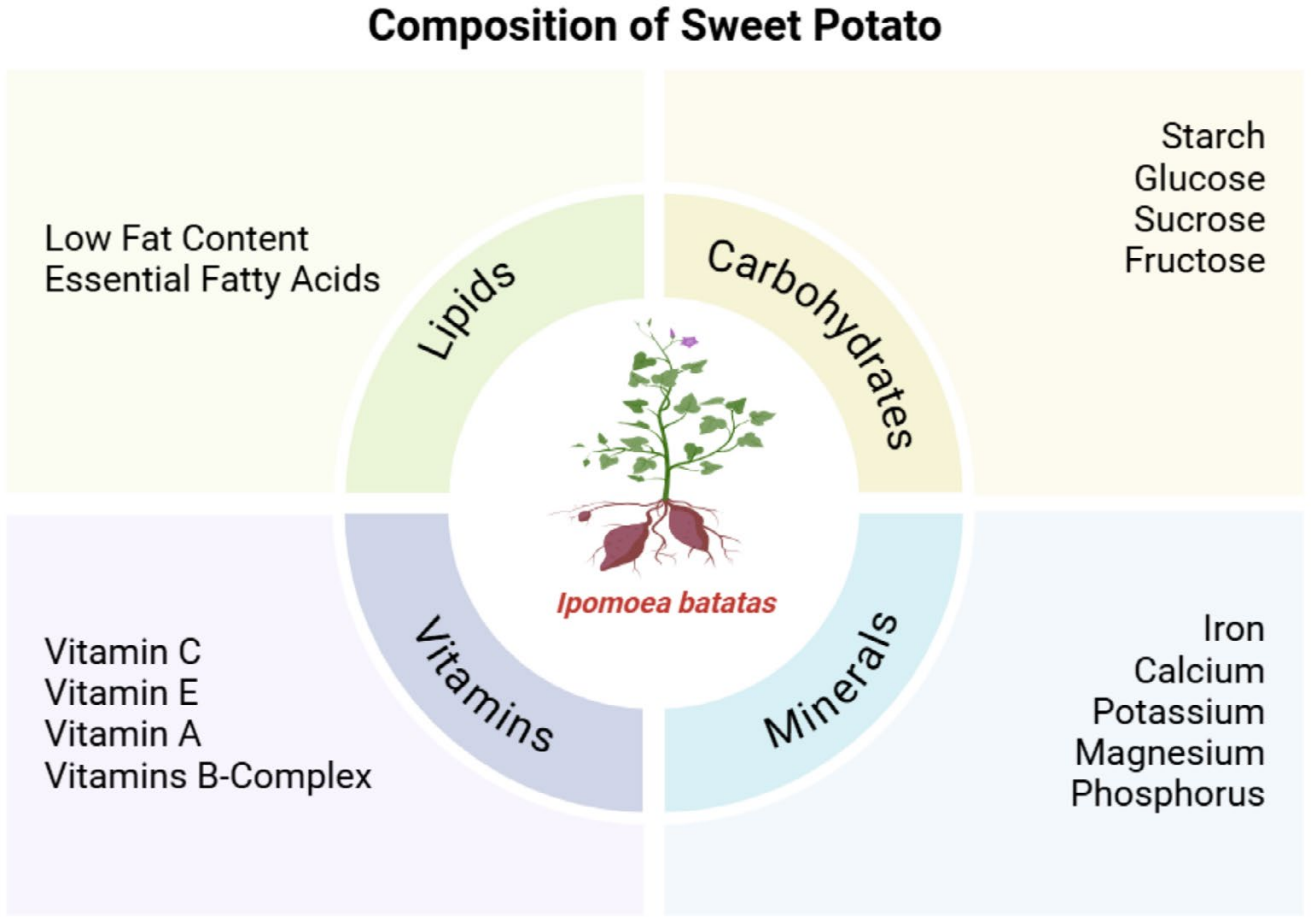
Nutritional composition of sweet potato.

*Ipomoea batatas* , or sweet potatoes, is considered one of the most nutrient‐condensed and versatile root crops. These vegetables offer many benefits other than the well‐known anthocyanin content health‐promoting dietary advantages. Specifically, metabolic health, fiber, vitamins, and minerals are key to this concise review of nutritional profiles, function in improving food quality, and potential therapeutic applications. Sweet potatoes are valued for their anthocyanin content and are rich in vitamins, minerals, and nutritional fiber. These nutrients play a significant role in overall health and well‐being. Some of the most critical elements contained in sweet potatoes are rich in dietary fiber and bioactive compounds such as anthocyanins and flavonoids, which help promote heart health and aid digestion (Baky et al. 2022). Fibers, flavonoids, and anthocyanins maintain gut health, are vital for metabolic health, and help regulate blood sugar levels (De and De 2021; Baky et al. 2022). Figure 3 shows the nutritional composition of sweet potato.

Furthermore, this fiber also promotes fullness/satiety, which reduces overeating and assists in weight control. Sweet potatoes are rich in vitamin A in the form of beta‐carotene, which is essential for healthy skin, eyes, and immune structures. Sweet Potato also contains adequate amounts of vitamin C, an antioxidant that helps to improve skin health and immunological function. B vitamins, such as B6, are also found in sweet potatoes and are essential for energy metabolism and neurological system functions (Islam 2024; De and De 2021).

These tubers are rich in minerals, the most important of which is K. Potassium‐rich foods may help reduce blood pressure (Ellison and Terker 2015). Sweet potatoes also contain calcium and magnesium for healthy bones and muscles (Islam 2024). Owing to their low glycemic index, sweet potatoes, a rich cradle of carbohydrates, are an excellent option as a steady energy source, perfect for people with diabetes or anyone looking to regulate their blood sugar. Sweet potatoes contain complex carbohydrates, which are digested more slowly than simple carbohydrates and cause no increase in blood sugar (Corbitt, n.d.).

Owing to their rich nutrition, sweet potatoes are critical elements in refining the eminence of diets. When one talks about the health assistance provided by foods, the concept of NRF is indispensable, and an extraordinary nutritional density is observed in sweet potatoes. The number of healthy nutrients controlled by sweet potatoes subsidizes their extraordinary NRF scores. NRF scores are calculated based on the percentage of vital vitamins, minerals, fiber, and other bioactive substances in meals (Drewnowski and Burton‐Freeman 2020). The addition of sweet potatoes to a balanced diet may provide numerous health benefits including weight management, digestion, and immunity. Sweet potatoes have potential medical applications in addition to their role in general nutrition. Studies have suggested that the bioactive ingredients of sweet potatoes, including anthocyanins and carotenoids, may possess antioxidant and anti‐inflammatory properties, which can help manage chronic diseases, such as diabetes, heart disease, and obesity. Table 3 depicts the nutritional benefits and potential therapeutic use of sweet potatoes.

**TABLE 3:** Nutritional benefits and potential therapeutic use of sweet potatoes.

Aspect	Details	References
Nutrient composition	Rich in dietary fiber, vitamins (A, C, B6), minerals (potassium, magnesium), and anthocyanins with antioxidant properties	(Wang et al. 2016; Jiang et al. 2019)
Diet quality enhancement	Contributes to improved metabolic health by reducing oxidative stress and supporting gut microbiota	(Vlaicu et al. 2023; Chen et al. 2024)
Anti‐diabetic properties	Low glycemic index; contains bioactive compounds that modulate glucose metabolism and reduce insulin resistance	(Jiang et al. 2020; Sudhakar et al. 2021)
Anthocyanin bioavailability	Purple sweet potatoes contain protein‐bound anthocyanins, which enhance stability and bioavailability	(Herrera‐Balandrano et al. 2021; Martín et al. 2018)
Functional food applications	Incorporated in diabetic management programs and disease prevention strategies	(Elgabry et al. 2023; Amagloh et al. 2022)
Cardiovascular benefits	Reduces cholesterol levels and supports cardiovascular health through antioxidant and anti‐inflammatory properties	(Zhu et al. 2024; Priyadarshini et al. 2019)
Skin and eye health	Rich in beta‐carotene, enhancing skin elasticity and protecting against age‐related macular degeneration	(Mattoo et al. 2022; Gonzali and Perata 2020)
Immune system support	Boosts immunity through high levels of vitamin A and anthocyanins, which reduce inflammation and strengthen defense mechanisms	(Narwal et al. 2024; Teng et al. 2021)
Weight management	Low in fat and calories while being nutrient‐dense, making it a valuable addition to weight loss diets	(Dixit et al. 2023; Siddiqui et al. 2023)
Recipe integration	Easily integrated into daily diets as baked, mashed, or in soups and desserts, maximizing nutritional intake	(Islam 2024; Ebert 2022)
Cancer prevention	Antioxidant properties of anthocyanins and polyphenols help reduce cancer risk by neutralizing free radicals	(Drewnowski and Burton‐Freeman 2020; Amagloh et al. 2021)
Improved digestive health	High fiber content promotes gut health and prevents constipation	(De and De 2021; Sapwarobol et al. 2021)
Bioactive compound synergy	Combined with other foods (e.g., protein, fruits) to create synergistic effects that amplify health benefits	(Corbitt, n.d.; Eivers, n.d.)
Starch digestibility	Contains slowly digestible starch, beneficial for maintaining steady glucose levels	(Jiang et al. 2020; Huynh, n.d.)
Sustainability aspect	Sweet potatoes are resilient and sustainable crops that can address food security in developing regions	(Islam 2024; Amoanimaa‐Dede et al. 2020)

Sweet potatoes are a good food for patients with type 2 diabetes because they contain 16% DW of fiber, which is used to regulate blood glucose levels (Nitzke et al. 2024). Speedy blood sugar spikes are a significant problem in patients with diabetes, although their squat glycemic index prevents them from experiencing this problem (Corbitt, n.d.). Potassium attentiveness also helps regulate blood pressure, which benefits heart health. Sweet potatoes have high levels of anthocyanins and potent antioxidants, especially those with purple flesh. These chemicals help the body fight free radicals by reducing oxidative stress, which leads to chronic diseases. Because of their antioxidant activity, sweet potatoes are essential in an anticancer diet, and their association with a reduced risk of some types of cancer has also been linked to their antioxidant activity (Drewnowski and Burton‐Freeman 2020).

Chronic inflammation (“inflammaging”) contributes to many health problems such as diabetes, arthritis, aging, and heart disease (Li et al. 2023). The anti‐inflammatory properties of sweet potatoes, particularly those related to the anthocyanin content, may reduce these risks. Consumption of sweet potato can help reduce other signs of inflammation, thereby contributing to better overall health (Gonzali and Perata 2020). Sweet potatoes are beneficial for digestive health because of their high fiber richness. As a prebiotic, soluble fibers trigger the growth of healthy gut flora. This may reduce the risk of constipation, improve digestive health, and alleviate symptoms of irritable bowel syndrome (De and De 2021).

**FIGURE 4:**
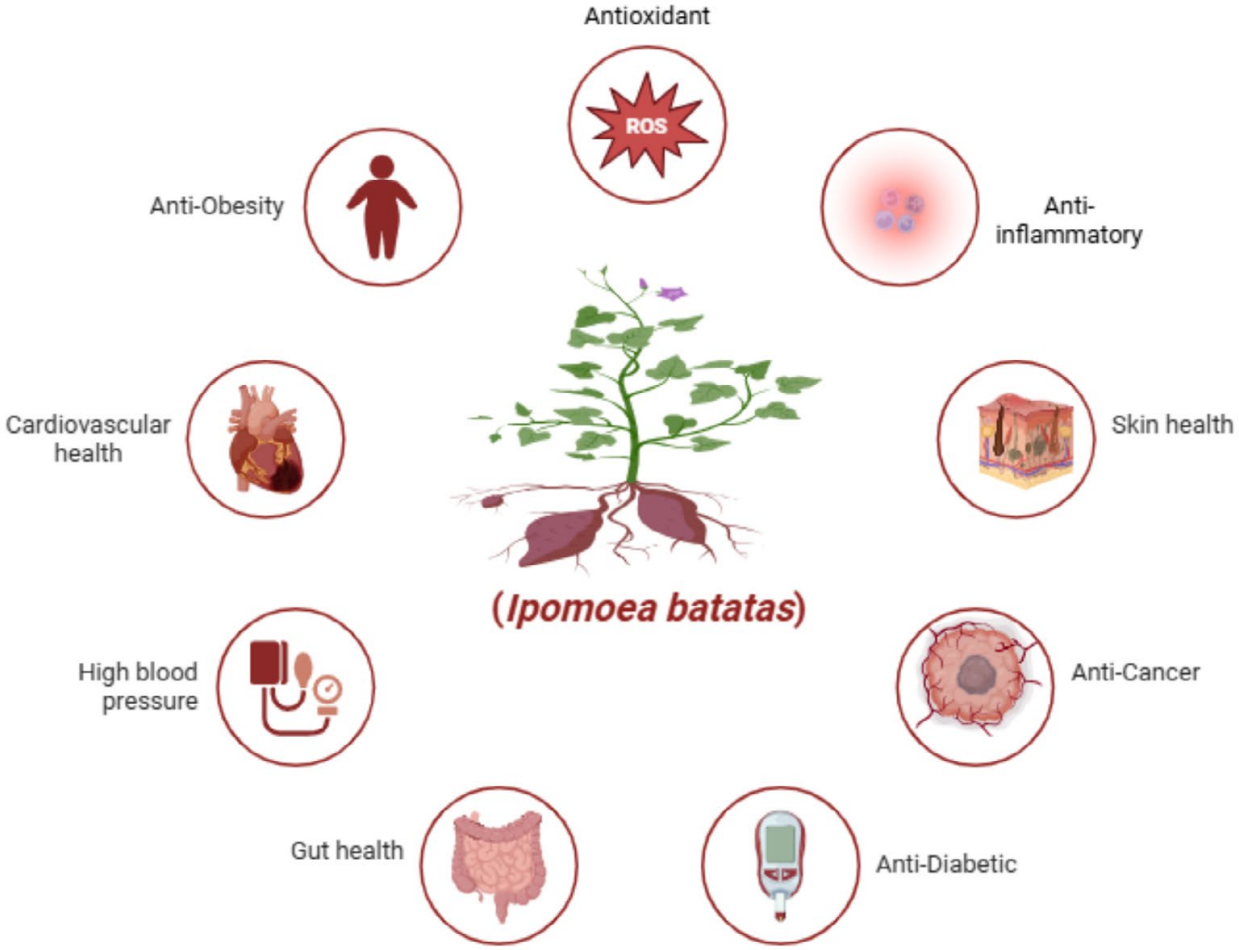
Therapeutic potential of sweet potato.

In addition, antioxidants in sweet potatoes may help to protect against oxidative impairment of the inner lining of the digestive tract. Sweet potatoes offer several health benefits. Anthocyanins are essential foods in a stable diet because they are rich in fiber, vitamins, minerals, and antioxidants. In addition to their role in fundamental nutrition, sweet potatoes can be used therapeutically for chronic diseases, especially those related to metabolism, such as diabetes, obesity, and cardiovascular diseases. As research advances, sweet potatoes may have additional therapeutic uses, underscoring their importance in encouraging long‐term health and well‐being. Figure 4 shows the therapeutic potential of sweet potato.

## 6 Mechanisms of Actions: Sweet Potatoes

### 6.1 Antioxidant Activity

Sweet potatoes have a well‐known antioxidant content, especially in purple flesh. Some bioactive substances contain anthocyanins, which offer such advantages and defenses against diseases that lead to cancer and heart and even neurological disorders by scavenging free radicals that decrease oxidative stress and antioxidant enzyme modulation. The specific mechanisms involved in the mechanism of action of anthocyanins, including the scavenging and activation of antioxidant enzymes, are discussed in this section. Oxidative stress occurs when the production of ROS and the body's ability to neutralize them with antioxidants are out of balance. ROS, including H_2_O_2_, $O_{2}^{\cdot -}$, and ·OH, damage DNA, proteins, and lipids, leading to aging and the etiology of many diseases. Anthocyanins are potent antioxidants that help protect the body by neutralizing ROS. The primary antioxidant activity of sweet potato anthocyanins is attributed to their ability to stabilize and neutralize free radicals by donating electrons or hydrogen atoms. The antioxidant functions of anthocyanins are closely related to their molecular structures. The delocalized electrons in their aromatic rings make anthocyanins excellent scavengers of free radicals. Because hydroxyl groups may form steady phenoxyl radicals upon donating a free radical to an electron, their incidence in the molecule further augments their free radical‐scavenging ability (Ma et al. 2020).

Purple sweet potato anthocyanin extracts had marked scavenging activities against several free radicals, such as DPPH (1,1‐diphenyl‐2‐picrylhydrazyl), ABTS (2,2′‐azino‐bis (3‐ethylbenzothiazoline‐6‐sulfonic acid)), and hydroxyl radicals, as cited in a landmark study by Jiao et al. (2012). Therefore, purple sweet potato anthocyanins are effective at reducing oxidative damage. Zhao et al. (2013) also found that anthocyanins from purple sweet potatoes have potent in vivo antioxidant activity in animal models of oxidative impairment, such as tert‐butyl hydroperoxide, which lowered oxidative stress indicators. Lipid peroxides, which are essential markers of oxidative stress, can also be scavenged by anthocyanins. By inhibiting lipid peroxidation caused by ROS, these compounds protect cell membranes and other tissues from damage (Teow et al. 2007). This antioxidative process protects the cardiovascular and other physiological systems of anthocyanins.

In addition to directly scavenging free radicals, anthocyanins support antioxidant defenses by activating endogenous antioxidant enzymes that are necessary to maintain cell redox balance. Such enzymes include glutathione peroxidase (GPx), catalase (CAT), and superoxide dismutase (SOD). The activation of these enzymes increases the ability of the body to degrade ROS and restore cells to equilibrium. The first defense mechanism against ROS is SOD, an enzyme that catalyzes the conversion of superoxide radicals ($O_{2}^{\cdot -}$) into O_2_ and H_2_O_2_. Inhibition of cell damage requires the conversion of superoxide radicals to less dangerous forms. Anthocyanins also increase SOD activity. Hwang et al. (2011) reported that purple sweet potato anthocyanins significantly increased SOD activity in oxidatively stressed liver cells, indicating that anthocyanins have the potential to activate crucial enzymes.

CAT, another vital antioxidant enzyme, decomposes hydrogen peroxide into oxygen and water, thereby protecting cells from the harmful effects of hydrogen peroxide accumulation. Grace et al. (2014) demonstrated that anthocyanin‐rich sweet potato extracts increase CAT activity in human hepatocytes, indicating that anthocyanins can enhance the activity of enzymes responsible for hydrogen peroxide detoxification. This elevated CAT activity lowers the potential cytotoxicity of hydrogen peroxide, which often results from cellular metabolism (Grace et al. 2014). Another mechanism by which anthocyanins reduce oxidative stress is the activation of GPx. GPx neutralizes lipid hydroperoxides and hydrogen peroxide using glutathione as the reducing agent. Jiao et al. (2012) showed that anthocyanins from purple sweet potatoes exhibited elevated GPx activity, which supplemented their antioxidative potency. In addition to these commonly known antioxidant enzymes, anthocyanins can affect the expression of other genes and proteins that are sensitive to oxidative stress. For instance, the anticancer properties of anthocyanins have been associated with the activation of nuclear factor erythroid 2‐related factor 2 (Nrf2), a transcription factor responsible for the expression of hundreds of antioxidant genes (Jiao et al. 2012). Cytoprotective enzymes, including glutathione metabolism and redox balance, are induced in response to Nrf2 activation (Garcia and Blesso 2021).

Anthocyanins activate the Nrf2 pathway, enhancing the body's resistance to oxidative stress and inducing antioxidant enzymes (Kano et al. 2005). In addition to their chemical structure, extrinsic factors, including food‐processing techniques, influence the antioxidant potential of anthocyanins in sweet potatoes. For instance, the potency and bioavailability of anthocyanins can be altered by boiling, steaming, and baking. Nonetheless, Wang et al. reported that gentle processing techniques such as steaming helped protect anthocyanins from the purple variety of sweet potatoes. This means that the variety of sweet potatoes and other intrinsic or extrinsic factors, such as cooking method, may influence the antioxidant activity of anthocyanins (Wang, Wu, et al. 2020). In addition, the antioxidant activities of anthocyanins from sweet potatoes may synergize with those of other bioactive compounds such as vitamins, carotenoids, and phenolic acids, enhancing their free radical scavenging and oxidative damage reduction. For example, purple‐fleshed sweet potatoes may exert a more significant effect on oxidative stress than anthocyanins or β‐carotene alone because of their combined occurrence (Teow et al. 2007).

Sweet potato anthocyanins, especially those from purple‐fleshed varieties, have excellent antioxidant activities through several mechanisms, including direct scavenging of free radicals and activation of antioxidant enzymes, such as GPx, CAT, and SOD. Oxidative stress is reduced, thereby protecting the tissues and cells from damage. The potential therapeutic benefits of anthocyanins in anticipating and handling oxidative damage‐related diseases, such as cancer, cardiovascular disease, and neurodegenerative disorders, are underscored by their ability to activate endogenous antioxidant defense mechanisms and moderate oxidative stress pathways. Understanding the complex processes through which these compounds exert their antioxidant effects will allow us to appreciate the health‐promoting properties of sweet potatoes and other high‐anthocyanin foods.

### 6.2 Cellular and Molecular Mechanisms of Anthocyanins in Sweet Potatoes

Anthocyanins, bioactive molecules in purple‐fleshed sweet potatoes, have been thoroughly studied for their antioxidant activity and ability to modulate oxidative stress at the molecular and cellular levels. These molecules have shown the ability to reduce oxidative damage to cellular structures by scavenging free radicals and adapting essential signaling pathways associated with inflammation and metabolism (Li et al. 2023).

Lipid peroxidation is a primary outcome of oxidative stress. Here, free radicals aggressively attack lipids in the cell membranes, causing damage and malfunction. The studies have proved that anthocyanins, especially those in purple‐colored sweet potatoes, significantly reduce lipid peroxidation. They act mainly by directly scavenging ROS, including hydroxyl radicals and superoxide anions. Several studies have demonstrated that anthocyanins can effectively inhibit lipid oxidation and protect the cell membranes from damage. For example, tert‐butyl hydroperoxide (TBHP), a known oxidative stress inducer, reduces lipid peroxidation in purple sweet potato anthocyanin extract‐treated liver cells (Hwang et al. 2011). These protective effects determine the integrity and functionality of cells. Anthocyanins also maintain their cellular structure by protecting proteins and other macromolecules from oxidative degradation. This is most evident in crucial metabolic regulation organs such as the muscles, pancreas, and liver. For instance, anthocyanins have been shown to increase the activity of endogenous antioxidant enzymes such as CAT and SOD (Kano et al. 2005).

Anthocyanins neutralize ROS and reduce oxidative stress by increasing the activity of these enzymes, which is crucial for the conservation of liver function and detoxification. Anthocyanins can shift redox balance in pancreatic cells by scavenging antioxidants. Two significant advantages of this effect are its role in preserving insulin sensitivity and supporting impaired metabolic processes in conditions such as diabetes (Zhao et al. 2013). Likewise, in muscle cells, anthocyanins increase muscle performance and durability by reducing the oxidative impairment of mitochondrial structures, which are used in energy manufacturing (Teow et al. 2007).

### 6.3 Mechanisms of Action in Key Organs

Detoxification occurs in the liver, and metabolic control is another system in which one of the organs and liver processes detoxifies phases 1 and 2 (Hodges and Minich 2015). Purple sweet potato anthocyanins have been shown to protect liver cells from oxidative damage by altering gene expression in the antioxidant defense pathways (Hodges and Minich 2015). SOD and CAT antioxidant enzymes are induced when anthocyanins specifically activate the nuclear factor erythroid 2‐related factor 2 (Nrf2) signaling pathway (Kim et al. 2015). Additionally, anthocyanins are believed to suppress pro‐inflammatory pathways, thereby reducing liver inflammation and promoting tissue repair (Hwang et al. 2011). The pancreas plays crucial roles in insulin synthesis and glucose homeostasis. It has been reported that anthocyanins in sweet potatoes protect pancreatic β‐cells by reducing inflammation and oxidative stress, factors that trigger the development of diabetes (Zhao et al. 2013).

Anthocyanins contribute to the maintenance of β‐cell function by reducing the antioxidant activity and improving insulin secretion and glucose control. Muscle tissue is susceptible to oxidative stress because of its high metabolic rate. Anthocyanins protect muscle cells by increasing cumulative antioxidant dimensions and reducing ROS levels, which may otherwise cause muscular fatigue and injury (Teow et al. 2007). By scavenging ROS, anthocyanins keep muscle fibers intact and enhance muscle recovery through subsequent exercise.

Other aspects include the impact of anthocyanins on molecular signaling cascades that regulate the metabolic mechanisms of oxidative stress and further determine specific regulatory pathways. One is the Nrf2 pathway, which regulates the transcriptional activity of antioxidant genes and the rate of cellular responses to oxidative stress. Anthocyanins cause activation of Nrf2, which leads to further expression of antioxidant enzymes‐ends guarding cell components from oxidative damage (Zhao et al. 2013). Furthermore, it has been shown that anthocyanins modulate inflammatory pathways by inhibiting the activation of nuclear factor‐kappa B (NF‐κB). This transcription factor controls the expression of pro‐inflammatory cytokines (Hwang et al. 2011).

Purple sweet potato anthocyanins have also recently been demonstrated to affect the AMPK pathway, which regulates metabolic and cellular energy. Zhu et al. (2010) reported that AMPK activation enhanced mitochondrial biogenesis and oxidative metabolism, potentially mitigating oxidative stress in cells (Zhu et al. 2010). The bioavailability and effectiveness of anthocyanins in sweet potatoes can be altered by cooking techniques. Several studies have shown that boiling, steaming, and roasting affect the content and antioxidant activity of anthocyanins in sweet potatoes (Liao et al. 2019). For example, the anthocyanin content of sweet potatoes is better preserved during boiling and steaming than during roasting, which results in the preservation of higher antioxidant activity. This makes cooking techniques even more important for complete exploitation of the health benefits of sweet potatoes. Anthocyanins from purple sweet potatoes have diverse cellular and molecular mechanisms that modify oxidative stress, lipid peroxidation, and cellular protection in crucial organs, such as the muscles, liver, and pancreas. These complexes activate critical antioxidant enzymes and pathways such as Nrf2 and AMPK to promote cellular health and metabolic balance. In addition, anthocyanin antioxidant activity is influenced by cooking methods and hence affects their bioavailability. These instruments interact supportively to support the perceived beneficial actions of anthocyanins, especially during disease processes characterized by metabolic perturbation and oxidative stress.

### 6.4 Impact on Oxidative Damage and Aging

Research has shown that anthocyanins in purple sweet potato ( *Ipomoea batatas* ) promote the antioxidant defense system and scavenge ROS to produce protective benefits. In vitro studies have shown that anthocyanins protect cellular components such as proteins, DNA, and lipid membranes from oxidative damage. For instance, purple sweet potato anthocyanins have been demonstrated to protect against cell membrane impairment by inhibiting lipid peroxidation, an essential indicator of oxidative stress (Kano et al. 2005).

Anthocyanins also protect the DNA and proteins from oxidative damage. For example, anthocyanin‐rich extracts from purple sweet potatoes have been used to mitigate protein carbonylation, which is often associated with cellular aging, and abrogate oxidative DNA damage (Zhao et al. 2013; Ye et al. 2010). These mechanisms preserve the integrity of cells and prevent oxidative damage, which accelerates aging. In addition, anthocyanins have been shown to stimulate the activity of the natural antioxidant enzymes GPx, CAT, and SOD, which eliminate free radicals and reduce the burden of oxidative impairment in cells (Shan et al. 2009). The activation of these enzymes is further evidence that anthocyanins can delay the process of aging by strengthening cell defenses against oxidative damage.

#### 6.4.1 Implications for Aging and Age‐Related Diseases

The antioxidative properties of anthocyanins have broad implications in aging and in the anticipation of age‐related diseases, ranging from cardiovascular diseases to neurological disorders and skin aging. Current research has shown that purple sweet potato anthocyanins can interfere with several aging pathways, including cellular senescence, autophagy, and inflammation, all of which are associated with age‐related diseases. Loss of cognitive function, a condition often associated with oxidative stress and neuroinflammation, is associated with aging. Studies have shown that purple sweet potato anthocyanins reduce oxidative stress in the brain and protect the neurons from damage. For example, Shan et al. (2009) found that anthocyanins in purple sweet potatoes improved cognitive deficiencies and decreased d‐galactose‐induced oxidative impairment in the brains of aged mice. Anthocyanins help maintain cognitive function by preventing inflammation and oxidative stress, which might delay the onset of neurodegenerative disorders, such as Alzheimer's disease (Shan et al. 2009).

Oxidative stress is the primary cause of deterioration of collagen and elastin fibers, which causes wrinkles and other manifestations of photoaging. Based on research conducted by Zhi et al. (2020), anthocyanins found in purple sweet potatoes have been established to protect human skin cells exposed to UVB light, which causes photoaging. Anthocyanins improve the skin's resistance to environmental stresses, sustain skin collagen, and reduce oxidative damage. This indicates that anthocyanin‐rich dietary supplements could be a conduit to prevent or at least slow the skin aging process (Zhi et al. 2020).

Purple sweet potato anthocyanin extracts have an enhanced life span in model organisms such as *Drosophila melanogaster* and *Caenorhabditis elegans* . As indicated by Han et al. (2021), purple sweet potato extract extends the lifespan of Drosophila by activating the autophagy pathway, which plays a role in maintaining and repairing cells. Autophagy improves the quality of cells and prolongs their lifespan by facilitating the degradation of harmful proteins and organelles (Han et al. 2021). Similarly, studies on *C. elegans* have demonstrated that fermented anthocyanin extracts decrease oxidative stress and activate cell defense mechanisms to show a pronounced anti‐aging effect (Zhao et al. 2021).

Inflammation is another significant factor leading to aging and age‐related diseases. Purple sweet potato anthocyanins have been found to suppress inflammatory pathways and reduce the expression of pro‐inflammatory cytokines, including TNF‐α and IL‐6. A study by Sun et al. demonstrated that purple sweet potato hue suppressed premature endothelial senescence by inhibiting the NLRP3 inflammasome, thus showing anti‐inflammatory activity. Anthocyanins may reduce chronic diseases, such as diabetes and cardiovascular disease, and are often associated with aging by exerting anti‐inflammatory effects (Sun et al. 2015).

The development of metabolic diseases such as obesity, diabetes, and cardiovascular diseases is also affected mainly by oxidative stress. Studies have shown that purple sweet potato anthocyanins increase insulin sensitivity, reduce oxidative stress, and inhibit fat‐induced apoptosis in model organisms, which can further improve metabolic health (Wang et al. 2016). These populations are particularly vulnerable because they are more susceptible to metabolic dysregulation. In summary, purple sweet potato anthocyanins have high antioxidant capabilities, reducing oxidative deterioration of proteins, lipids, and DNA. This results in the prevention of age‐related diseases. These effects may make anthocyanins significant in reducing inflammation, improving cellular repair systems, decreasing oxidative stress, and preventing or delaying aging, while elongating life expectancy. Further research on their mechanisms and treatment potential may provide important insights into therapies for age‐associated health.

### 6.5 Anthocyanins in Diabetes Prevention and Management

A key characteristic of the chronic metabolic disease type 2 diabetes, also known as T2D, is impaired insulin responsiveness by the body's cells and dysfunction in the β‐cells of the pancreas; accordingly, blood glucose levels tend to increase and manifest as this illness. Cardiovascular disease, neuropathy, and retinopathy are long‐term complications that may result from the inability to regulate blood glucose levels (Ayeleso et al. 2016).

Many factors, including genetic predisposition, lifestyle factors such as poor diet and physical inactivity, and environmental influences, play an intricate role in the pathophysiology of T2D. Oxidative stress and inflammation are crucial factors in T2D development. ROS generation beyond the body's antioxidant level leads to oxidative stress, causing damage to cellular constituents such as proteins, DNA, and lipids. According to Ayeleso et al. (2018), this damage can exacerbate insulin resistance by altering insulin signaling. Chronic low‐grade inflammation decreases insulin sensitivity and increases insulin resistance (Ayeleso et al. 2018), thereby significantly influencing the onset and progression of T2D, as concluded by Ayeleso et al. (2016). Managers and preventers can reduce the burden of T2D. Management starts with changing lifestyles, as dietary changes are considered the most significant factor in managing the condition. However, other medications, such as insulin, are commonly used to decrease blood glucose levels. Dietary approaches that increase antioxidant and anti‐inflammatory food intake, such as sweet potatoes, help reduce hyperglycemia and improve general metabolic health (Ayeleso et al. 2016).

Essential to controlling and preventing T2D are dietary treatments that address the root causes of insulin resistance and reduce oxidative stress. A growing body of research supports the consumption of functional foods such as sweet potatoes, which contain bioactive chemicals that can reduce the onset of diabetes. Sweet potatoes contain carotenoids, phenolic compounds, and anthocyanins, which have powerful anti‐inflammatory and antioxidant activities, especially in purple‐ and orange‐fleshed cultivars. Researchers are said to increase sensitivity to insulin by decreasing oxidative stress, while high fiber content helps regulate blood glucose levels by reducing glucose absorption (Luo et al. 2021a).

Sweet potatoes are also ideal for those with T2D because they have a minor GI compared to foods with an advanced GI, which means that they take longer to affect blood glucose levels (Mohanraj and Sivasankar 2014). Preclinical studies have also revealed that sweet potatoes positively affect diabetes mellitus. Animal models have demonstrated the vigorous antihyperglycemic activity of sweet potato extracts.

Some studies have revealed that blood glucose levels are reduced in diabetic mice and that insulin sensitivity is improved by sweet potato extract (Akhtar et al. 2018; Kamal et al. 2018). Anthocyanins, the pigments that give some sweet potato cultivars a purple color, have been shown to be noteworthy bioactive compounds that support anti‐diabetic benefits. Anthocyanins have been shown to alter glucose metabolism by regulating enzymes involved in carbohydrate digestion and absorption of carbohydrates (Arisanti et al. 2023).

Sweet potatoes may thus contribute to reducing the complications associated with diabetes in addition to improving glucose metabolism. For example, anthocyanins from purple sweet potatoes have been shown to improve blood glucose regulation and reduce oxidative stress markers (Mi et al. 2024). Furthermore, these substances facilitate gut microbial restoration, which plays an essential role in metabolic health and T2D therapy (Mi et al. 2024). Sweet potatoes also contain other nutrients, including vitamins, minerals, and fibers, which support the functioning of the immune system and general well‐being. Polyphenols and other bioactive compounds in sweet potatoes are suspected to be responsible for the reduced inflammatory processes that damage pancreatic β‐cells and exacerbate insulin resistance (Naomi et al. 2021). In addition, consuming sweet potatoes may alter metabolic pathways. This will help in the management and prediction of T2D. Several clinical studies have explored the potential of sweet potatoes as constituents of dietary therapies for T2D. For instance, in one study, in which purple sweet potato tuber extract was administered to participants with T2D, blood glucose levels were significantly lowered. This improves glycemic control (Mahadita et al. 2016).

An alternative study revealed that sweet potato leaf polyphenols alleviated hyperglycemia in diabetic rats, suggesting that sweet potato root and leaf parts could be therapeutically rummaged (Luo et al. 2021a). T2D is an intricate metabolic disease that is closely related to inflammation and oxidative stress. Dietetic treatment, including functional foods such as sweet potatoes, is a promising strategy for the regulation and prevention of T2D. Sweet potatoes have anti‐inflammatory and antioxidant properties, particularly those of anthocyanins and other bioactive compounds, as well as improved insulin sensitivity, reduced oxidative damage, and improved glucose metabolism. A further indication of the blood glucose control ability of sweet potatoes is their high fiber content and low glycemic index. A growing body of research on this topic proposes that sweet potatoes hold a beneficial nutritional aid in the fight against T2D, presenting an easy and natural approach to ornamental metabolic health.

*Ipomoea batatas* , commonly known as sweet potatoes, is highly recognized for its various health benefits, particularly in assisting with the management of various metabolic disorders such as diabetes. Anthocyanins are among the bioactive compounds responsible for the anti‐diabetic activity of sweet potatoes. This review discusses the mechanisms involved in enhancing insulin sensitivity, glucose metabolism, and the expression of genes related to insulin receptors and glucose transport. We also explored the potential of anthocyanins to modulate carbohydrate‐metabolizing enzymes. Anthocyanins are water‐soluble pigments found in the flesh of a variety of sweet potatoes with purple flesh. These substances have been shown to improve glucose metabolism and insulin sensitivity through several metabolic pathways (Khoo et al. 2017).

Anthocyanins exert anti‐diabetic effects by improving insulin signaling, reducing oxidative stress, and enhancing the function of pancreatic β‐cells responsible for insulin production. Arisanti et al. demonstrated that anthocyanins can modulate significant metabolic pathways involved in glucose homeostasis. Anthocyanins enhance glucose uptake by peripheral tissues and increase insulin receptor activity, thereby improving the overall efficiency of glucose metabolism (Arisanti et al. 2023). As insulin resistance is a hallmark of T2D, it is beneficial for those afflicted with the disease. Moreover, purple sweet potato anthocyanins have been shown to induce insulin sensitivity by altering the expression of genes related to glucose metabolism. Sweet potato anthocyanins have decreased levels of pro‐inflammatory cytokines such as TNF‐α, which are usually elevated in individuals with diabetes and eventually cause insulin resistance (Dutta 2015). Anthocyanins positively affect glucose sensitivity (Dutta 2015).

The glucose transporter 4, or GLUT4, is the primary transporter that facilitates glucose uptake into muscles and adipose tissues in response to insulin (Wang, Wang, et al. 2020). Glucose transport is considered to be an essential event in the regulation of blood glucose levels. Anthocyanins regulate GLUT4 expression, thereby enhancing glucose homeostasis. In another experiment, Ayeleso et al. (2018) demonstrated that orange‐fleshed sweet potato extracts altered GLUT4 expression in myotubes of C2C12 cells, resulting in insulin resistance. Consequently, sweet potato anthocyanins may increase GLUT4 gene expression, boosting the amount of glucose entering cells, especially the muscle cells. This process improves glucose metabolism (Ayeleso et al. 2018). This finding supports the hypothesis that anthocyanins can increase insulin sensitivity by boosting GLUT4 translocation to the cell membrane once insulin signaling is initiated. Anthocyanins may also influence insulin receptor expression. Akhtar et al. (2018) found that sweet potato extracts may improve insulin receptor activity in rats with alloxan‐induced diabetes. This increase in insulin receptor function improves insulin signaling and glucose uptake. Thus, anthocyanins enhance insulin signaling pathways by increasing insulin receptors and their sensitivity (Akhtar et al. 2018).

Another important mechanism through which sweet potato anthocyanins exert their anti‐diabetic effects is the inhibition of enzymes responsible for carbohydrate digestion. Multifaceted carbohydrates are broken down into humbler sugars by indispensable enzymes, such as α‐amylase and α‐glucosidase, which are later captured into circulation. It is common for people with diabetes to experience quick spikes in blood glucose levels following meals, which can be prevented by inhibiting enzymes. Several studies have reported that anthocyanins from sweet potatoes inhibit certain digestive enzymes from performing their functions. For instance, Ayeleso et al. (2024) identified that extracts of orange‐fleshed sweet potatoes could efficiently inhibit α‐amylase and α‐glucosidase activities, potentially reducing the rate of glucose absorption and carbohydrate digestion. Such an effect is significant for managing T2D as it may aid in better postprandial glucose administration (Ayeleso et al. 2024).

The inhibition of carbohydrate‐degrading enzymes is also responsible for lowering the glycemic index of sweet potato‐based diets. Such foods are essential because they result in a gradual increase in blood sugar levels. Sweet potatoes help to adjust glucose absorption by inhibiting such enzymes, thereby lowering the risk of hyperglycemia, a common fear among patients with diabetes (Kamal et al. 2018). Sweet potato anthocyanins play significant roles in diabetes management in several ways. Anthocyanins improve glucose metabolism and insulin sensitivity by upregulating GLUT4 expression and enhancing insulin receptor function. Moreover, anthocyanins regulate the absorption of glucose by inhibiting digestive enzymes such as α‐amylase and α‐glucosidase. Thus, anthocyanin‐rich purple‐fleshed sweet potatoes may be a promising dietary intervention for diabetes management. Future studies should focus on identifying the molecular mechanisms involved in the action of anthocyanins in diabetes management and their potential therapeutic applications.

### 6.6 Anti‐Inflammatory Properties

Chronic low‐grade inflammation has been shown to play a critical role in the development and pathogenesis of T2D, obesity, insulin resistance, and other metabolic diseases (“inflammaging”) (Hossain, Wazed, Asha, Amin, and Shimul 2025; Hossain, Wazed, Asha, Hossen, et al. 2025). Bioactive substances, such as anthocyanins, are polyphenolic compounds with bulky magnitudes in colored sweet potatoes, especially purple potatoes, and are crucial in regulating inflammation. This study discusses the potential therapeutic use of anthocyanins in the treatment of insulin resistance and the mechanisms involved in attenuating chronic inflammation. Anthocyanins are highly potent anti‐inflammatory and antioxidant compounds that can reduce inflammation in a series of organs, including the muscle, liver, and adipose tissues, which are potent contributors to insulin resistance. In various studies, purple anthocyanins from sweet potatoes have been shown to reduce the expression of pro‐inflammatory cytokines and markers such as NF‐kappa B, IL‐6, and TNF‐α, which are recognized to play a protective role in the pathophysiology of IR (Zhang et al. 2023; Belwal et al. 2017).

Anthocyanins neutralize ROS known to initiate inflammatory processes; they also suppress the progression of NF‐κB, an important regulator that promotes inflammation by curbing oxidative stress (Vendrame and Klimis‐Zacas 2015). Thus, pro‐inflammatory cytokines such as TNF‐α and IL‐6 were upregulated. NF‐κB is responsible for most insulin resistance triggered by obesity through its induction during metabolic stress (Panchal et al. 2022). Studies have shown that purple sweet potato anthocyanins prevent the activation of an additional critical signaling pathway involved in inflammation, the MAPK pathway (Li et al. 2018). Oxidative stress activates the MAPK pathway, leading to the expression of inflammatory cytokines. By inhibiting the NF‐κB and MAPK pathways, anthocyanins can prevent insulin resistance and decrease systemic inflammation (Ngamsamer et al. 2022).

Anthocyanins have been shown to suppress the expression of SOCS3 and galectin‐3, which are involved in inflammatory activities in insulin‐resistant tissues, while also altering NF‐κB and MAPK. For example, by downregulating SOCS3 and galectin‐3, anthocyanins from purple sweet potatoes decrease hippocampal insulin resistance in rats fed a high‐fat diet (Qin et al. 2019). This progression is particularly relevant for understanding how anthocyanins mitigate the metabolic alterations caused by inflammation in organs other than the liver, such as the brain.

Recent studies have highlighted the role of gut microbiota in modulating inflammation and metabolic health. Anthocyanins have been demonstrated to influence gut microbiota composition by promoting beneficial gut bacteria, which may reduce inflammation and enhance glucose homeostasis (Chen, Cao, et al. 2022). This finding is significant because dysbiosis, or disparity in gut microbiota, is increasingly recognized as a contributor to insulin resistance and universal inflammation. A series of inflammatory markers highly regulate the development and progression of insulin resistance. These include TNF‐α, IL‐6, and C‐reactive protein (CRP) levels. Studies have shown that anthocyanins modify these markers, and this reduction in inflammation increases insulin sensitivity.

TNF‐α is the most potent pro‐inflammatory cytokine that contributes to the development of insulin resistance. Binding to the insulin receptor substrate‐1 disrupts insulin signaling and impairs glucose metabolism. It has been proven that anthocyanins prevent TNF‐α from being secreted from several cell types, such as macrophages and adipocytes, thereby inhibiting systemic inflammation and enhancing insulin sensitivity (Franco‐San Sebastián et al. 2023). Similarly, anthocyanins create an anti‐inflammatory environment that improves glucose metabolism by suppressing IL‐6, a cytokine associated with insulin resistance (Kozłowska and Dzierżanowski 2021). NF‐κB is a crucial regulator of inflammation that controls the expression of numerous pro‐inflammatory genes, including IL‐6 and TNF‐α. It has been established that anthocyanins inhibit NF‐κB activation, which prevents the transcription of these inflammatory cytokines and reduces universal inflammation (Santamarina et al. 2023) in obesity and metabolic disorders, where chronic inflammation within the adipose tissue intensifies insulin resistance. Therefore, inhibition of NF‐κB expression is critical. CRP is another marker of inflammation commonly found in individuals with insulin resistance. Another approach through which anthocyanins may positively contribute to inflammation reduction and improve metabolic health may be through anti‐inflammatory effects that, in any case, have been proven to decrease CRP levels (Lee et al. 2017; Zhao et al. 2013). Evidence for the avoidance of diabetes recommends consuming 1–2 servings (100–200 g) of purple sweet potato per day to benefit from the health assistance of its anthocyanins and flavonoids, comprising glucose regulation and antioxidant capability (Zhao et al. 2013).

Anthocyanins have been demonstrated to improve insulin sensitivity and reduce inflammation in various preclinical and clinical studies. Santamarina et al. (2023) showed that anthocyanin‐rich dietary supplements significantly decrease inflammatory markers in obese individuals and improve insulin sensitivity. Other studies have also used purple sweet potato extract in animal models, where it has been shown to significantly reduce inflammatory indicators and improve glucose metabolism, thus highlighting the potential therapeutic benefits of anthocyanins in preventing and managing insulin resistance and metabolic disorders (Mi et al. 2024).

In addition to their direct effect on inflammation, anthocyanins stimulate adiponectin secretion and enhance insulin receptor activation, thereby improving metabolic health. According to Chen, Cao, et al. (2022) and Chen, Kortesniemi, et al. (2022), these properties are crucial for improving insulin sensitivity and delaying the onset of T2D. Hence, there is great potential for natural treatment with sweet potato anthocyanins in patients with insulin resistance and consequent chronic diseases (Chen, Cao, et al. 2022; Chen, Kortesniemi, et al. 2022). The key aspects of insulin resistance and metabolic disease could be decreased by anthocyanins, particularly anthocyanins, in purple sweet potatoes. It may provide a unique diet‐based intervention to enhance the metabolism of the human body in multiple ways, such as its role as an antioxidant by controlling inflammatory pathways, such as NF‐κB and MAPK, or by regulating other key inflammation markers, such as TNF‐α and IL‐6. Further research, including clinical trials, is needed to fully understand the therapeutic potential of anthocyanins in obesity and insulin resistance.

## 7 Clinical Evidence and Studies: Sweet Potatoes

*Ipomoea batatas* , or sweet potatoes, has been a staple food for a long time and has been recognized as healthy. Evidence suggests that they may be suitable controllers for the treatment of diabetes. Sweet potatoes and anthocyanins at high concentrations in purple‐fleshed types have shown potential anti‐diabetic effects in both clinical trials and animal models. Several clinical studies have investigated the effects of sweet potato on insulin resistance and hyperglycemia. This is because sweet potatoes have high fiber content, thus making them rich in several bioactive compounds like anthocyanins, making it possible for them, among other things, to cause significant reductions in blood glucose levels and recover insulin sensitivity, as discovered in a systematic review done by Arisanti et al. (2023).

In particular, purple sweet potatoes have shown promise in improving glycemic control in human subjects with type 2 diabetes and animal models. For example, Mi et al. (2024) reported that through changes in gut microbiota and augmented insulin sensitivity, anthocyanins from purple sweet potatoes restored normal blood glucose levels in type 2 diabetic mice. Sweet potatoes may also inhibit or reduce diabetes‐related disorders, such as inflammation and oxidative stress, which are the main causes of diabetic retinopathy and other microvascular issues. Table 4 depicts the preventive effects of sweet potatoes on diabetes.

**TABLE 4:** Preventive effects of sweet potatoes on diabetes.

Study type	Model/Subjects	Mode of action	Effects	References
Preclinical	Alloxan‐induced diabetic rats	Inhibition of carbohydrate‐metabolizing enzymes	Reduced fasting blood glucose levels, improved lipid profile	(Ayeleso et al. 2024; Akhtar et al. 2018)
Preclinical	Streptozotocin (STZ)‐induced rats	Antioxidant activity of anthocyanins, modulation of gut microbiota	Improved glycemic control, normalized gut microbiota	(Mi et al. 2024; Gofur et al. 2024)
Preclinical	STZ‐induced Sprague–Dawley rats	Anti‐inflammatory effects via NF‐κB signaling pathways	Amelioration of diabetic retinopathy	(Hisamuddin et al. 2023)
Preclinical	Type 2 diabetic mice	Activation of hepatic glucose metabolism pathways	Amelioration of hyperglycemia, increased insulin sensitivity	(Jiang et al. 2020)
Preclinical	Diabetic C2C12 cells	Activation of PI3K/Akt pathways	Improved insulin signaling	(Shyur et al. 2021)
Preclinical	STZ‐induced rats	Regulation of oxidative stress and diabetic genes	Lower oxidative stress, better glycemic control	(Ayeleso et al. 2018)
Preclinical	Diabetic mice	Upregulation of GLP‐1 secretion	Reduced blood glucose levels	(Tsuda 2015)
Preclinical	Alloxan‐induced rats	Antioxidant effects of bioactive compounds	Improved glucose and lipid metabolism	(Refaat et al. 2020)
Clinical	Type 2 diabetic patients	Modulation of postprandial glucose levels	Significant reduction in fasting blood sugar and HbA1c	(Corbitt, n.d.; Ludvik et al. 2004)
Clinical	Type 2 diabetic subjects	Reduction of oxidative stress	Improved glycemic control and reduced inflammatory markers	(Mahadita et al. 2016)
Preclinical	Insulin‐resistant C2C12 cells	Regulation of insulin resistance genes	Amelioration of insulin resistance	(Ayeleso et al. 2018)
Preclinical	High‐fat diet mice	Modulation of SOCS3 and galectin‐3 expression	Improved hippocampal insulin resistance	(Qin et al. 2019)
Preclinical	Mice with metabolic disorders	Anthocyanin‐mediated anti‐inflammatory activity	Reduced chronic low‐grade inflammation, enhanced insulin sensitivity	(Zhang et al. 2023; Belwal et al. 2017)
Preclinical	Type 2 diabetic mice	Modulation of gut microbiota	Restoration of gut microbiota balance, normalized blood glucose	(Mi et al. 2024)
Preclinical	Alloxan‐induced rats	Selenylation of polysaccharides	Enhanced antioxidant and anti‐diabetic effects	(Yuan et al. 2017)
Clinical	Type 2 diabetic patients	Regulation of glucose metabolism	Significant decrease in fasting glucose and HbA1c levels	(Corbitt, n.d.)
Preclinical	Diabetic C2C12 cells	Reduction of oxidative stress and inflammation	Enhanced antioxidant activity, reduced insulin resistance	(Ayeleso et al. 2018)
Clinical	Type 2 diabetic patients	Bioactive anthocyanins	Reduced oxidative stress markers, improved glycemic parameters	(Ludvik et al. 2004)
Preclinical	Mice with insulin resistance	Downregulation of inflammation‐related pathways	Enhanced insulin sensitivity	(Li et al. 2018)
Preclinical	Type 2 diabetic rats	Modulation of hepatic insulin pathways	Improved glucose tolerance and reduced fasting blood glucose	(Luo et al. 2021a)

According to Naomi et al., studies have demonstrated the antioxidant activity of sweet potato extracts. Scientists have investigated the potential beneficial effects of sweet potatoes in the management of hyperglycemia and dyslipidemia, especially in preventing diabetic retinopathy (Naomi et al. 2021). Ayeleso et al. revealed that cells extracted from orange‐fleshed sweet potatoes exhibited reduced oxidative stress and altered genes related to T2D (Ayeleso, n.d.; Ayeleso et al. 2024). Animal studies have provided concrete evidence of the anti‐diabetic properties of sweet potatoes. Akhtar et al. established that in rats with alloxan‐induced diabetes, the anti‐diabetic benefit of sweet potato extract improved lipid profiles and dramatically reduced blood glucose (Akhtar et al. 2018).

Ayeleso et al. (2024) also reported that extracts from orange‐fleshed sweet potatoes blocked the enzymes that metabolize carbohydrates, thus helping reduce blood glucose levels. These results suggest that sweet potatoes could have an anti‐diabetic effect through several mechanisms, including antioxidant activity and enzyme inhibition (Ayeleso et al. 2024). Preclinical and clinical studies have shown that sweet potatoes, specifically those rich in anthocyanins, could be very useful in controlling diabetes by reducing insulin resistance, improving glycemic control, and averting impediments associated with the disease. The beneficial effects of sweet potatoes on oxidative stress and inflammation are two significant issues encountered in patients with diabetes and are associated with their bioactive components, such as anthocyanins. Sweet potatoes can therefore be considered an essential dietary therapy for managing diabetes. Table 5 depicts the anthocyanin mechanism of action in diabetes management.

**TABLE 5:** Anthocyanin mechanism of action in diabetes management.

Mechanism of action	Evidence‐based intervention	References
Improving insulin sensitivity	Purple sweet potato anthocyanins enhance insulin sensitivity by activating PI3K/Akt pathway	(Akhtar et al. 2018; Shyur et al. 2021)
	White sweet potato extract regulates TNF‐α‐induced insulin resistance	(Shyur et al. 2021; Shih et al. 2020)
	Anthocyanin‐rich diets increase GLUT4 expression in muscle tissues	(Guo and Ling 2015; Godyla‐Jabłoński et al. 2024)
Enhancing glucose metabolism	Anthocyanins from purple sweet potato regulate hepatic glucose metabolism by reducing gluconeogenesis enzymes	(Jiang et al. 2020)
	Purple sweet potato polyphenols reduce fasting glucose levels in T2DM mice	(Luo et al. 2021b; Mi et al. 2024)
	Sweet potato anthocyanins modulate hepatic PEPCK expression, a key enzyme in gluconeogenesis	(Gofur et al. 2019)
Modulating enzymes in carbohydrate digestion	Orange‐fleshed sweet potato extract inhibits alpha‐amylase and alpha‐glucosidase activity	(Ayeleso et al. 2024)
	Purple sweet potato anthocyanins reduce postprandial hyperglycemia via enzyme inhibition	(Wang et al. 2016; Tsuda 2015)
	Carbohydrate metabolism regulated by orange‐fleshed sweet potato in diabetic rats	(Kamal et al. 2018)
Reducing oxidative stress	Anthocyanins from sweet potatoes enhance antioxidant enzyme activity, reducing oxidative stress in T2DM models	(Ayeleso et al. 2018; Franco‐San Sebastián et al. 2023)
	Purple sweet potato extract decreases malondialdehyde levels in diabetic patients.	(Mahadita et al. 2016)
	Phenolic compounds in sweet potato leaves combat oxidative stress in diabetic mice	(Luo et al. 2021b)
Anti‐inflammatory effects	Purple sweet potato anthocyanins inhibit NF‐κB and MAPK signaling, reducing inflammation	(Vendrame and Klimis‐Zacas 2015; Panchal et al. 2022)
	Anthocyanins reduce obesity‐induced inflammation linked to T2DM.	(Ngamsamer et al. 2022; Kozłowska and Dzierżanowski 2021)
	Sweet potato polyphenols downregulate inflammatory cytokines in diabetic models.	(Boukhers et al. 2023)
GLUT4 and insulin receptor regulation	Anthocyanins stimulate GLUT4 translocation in muscle cells.	(Ayeleso et al. 2018; Bhatt et al. 2021)
	Protein‐bound anthocyanins improve insulin receptor signaling in hepatic cells.	(Jiang et al. 2020)
	Sweet potato bioactives increase GLUT4 mRNA expression in C2C12 myotubes	(Ayeleso et al. 2018; Shyur et al. 2021)
Gut microbiota modulation	Purple sweet potato anthocyanins normalize gut microbiota, indirectly improving glucose metabolism	(Mi et al. 2024; Tsuda 2015)
	Fiber and polyphenols from sweet potatoes promote beneficial gut microbiota shifts	(Chen, Cao, et al. 2022; Zhang et al. 2023)
Cytokine regulation in T2DM	Sweet potato anthocyanins downregulate SOCS3 and Galectin‐3, critical mediators in insulin resistance	(Qin et al. 2019)
	Purple sweet potato color reduces inflammatory markers in T2DM	(Liu et al. 2024)
	Sweet potato bioactives improve pancreatic islet regeneration	(Mahadita et al. 2016; Shih et al. 2020)

## 8 Incorporating Sweet Potatoes in Diet

*Ipomoea batatas* , or sweet potatoes, are highly valued for their potential culinary uses and nutritional benefits. Among other reasons, their fiber content is high, glycemic index is low, and they contain several bioactive components that are beneficial for diabetes. This chapter evaluates the functional food potential of sweet potatoes in diabetes treatment and prevention, as well as their practical addition to anti‐diabetic diets. Sweet potatoes can be consumed in many ways and can easily be integrated into a diabetes‐friendly diet. Subsequent concepts provide feasible ways to incorporate sweet potatoes while increasing their nutritional content; roasting or baking sweet potatoes is one of the calmest and healthiest ways to consume them. Nutrients are retained during cooking; thus, the result is a natural, sweet, and filling meal. Sliced sweet potatoes, for example, make for a healthy side dish or a main course; brush olive oil on them, then roast at 400°F for 30–40 min until tender (Corbitt, n.d.).

The results from diabetic mouse studies indicate that the daily consumption of 100–150 g of purple sweet potato is capable of controlling blood glucose and even preventing diabetes (Zhao et al. 2013). Compared to boiling, this method has a subordinate glycemic effect. Steamed or boiled sweet potatoes can be mashed into creamy and delicious dishes by using unsweetened almond milk or a small amount of olive oil. It is an excellent substitute for mashed potatoes because it has a much lower glycemic index than that of mashed potatoes. Sweet potato mash can be added to casseroles, soups, and other side dishes. Sweet potato fries are healthier because they are baked in an oven and contain no oils. It is suitable for a diabetic diet because its natural glycemic index is lower than that of regular potatoes. To increase flavor, herbs such as thyme or rosemary are more appropriate than adding extra sugar (Corbitt, n.d.).

Sweet potatoes add natural sweetness and soak other flavors when chopped and added to soups and stews. Sweet potatoes subsidize filling high‐fiber meals that may control blood sugar when accompanied by low‐glycemic vegetables, such as beans and leafy greens (Strugała et al. 2019). Boil or steam, mash, and add them to your smoothies for a high‐quality drink. These are well paired with berries, spinach, low‐fat yogurt, and other dishes. This is an excellent strategy for controlling blood sugar spikes against the consumption of healthy foods such as vitamins, fiber, and antioxidants (Mi et al. 2024). Diced sweet potatoes may be present in roasted and boiled salads. Collectively with bitter greens such as kale or arugula, inherent sweetness creates a delicious and balanced dish. Such a salad may provide an excellent lunch or side dish option for patients with diabetes, owing to the high‐fiber and low‐glycemic carbs it contains (Jiang et al. 2020).

### 8.1 Sweet Potatoes as a Functional Food

Sweet potatoes have great potential as functional foods for diabetes treatment plans. They are replete with fiber, complex carbohydrates, and other nutrients such as potassium and vitamin A, which contribute to overall health. Sweet potatoes contain noteworthy amounts of fiber, which have been shown to reduce the increase in blood glucose following a meal by inhibiting the preoccupation of glucose into the bloodstream (Gofur et al. 2024). Moreover, sweet potatoes are digested and absorbed more slowly than high‐glycemic index foods owing to their low GI. For type 2 diabetics, this slow release of glucose helps maintain stable blood glucose levels (Corbitt, n.d.).

Studies have shown that substituting high‐glycemic foods with low‐glycemic foods such as sweet potatoes, glycemic management, and insulin sensitivity in diabetic patients can be improved (Strugała et al. 2019). In addition to blood sugar regulation, sweet potatoes contain antioxidants, such as beta‐carotene, among other phenolic compounds, that inhibit the increase in oxidative stress implicated in the onset of diabetes‐related issues, such as neuropathy and cardiovascular disease (Arisanti et al. 2023). These antioxidants prevent chronic diabetes‐related diseases by eliminating free radicals from the body. Sweet potatoes have anti‐inflammatory properties, which may alleviate diabetes. Chronic inflammation generally worsens insulin resistance, which is often observed in diabetic patients. This can be counteracted by the high content of anti‐inflammatory compounds in sweet potatoes, which enhance insulin action and reduce the risk of complications (Mi et al. 2024). Besides having an advantageous effect on disease control, sweet potatoes can avoid the risk of several chronic diseases. Anthocyanins represent a powerful armor against oxidative stress because of their high antioxidant content, which prevents many diseases associated with oxidative stress, obesity, heart diseases, and cancer (Dwivedi et al. 2022).

Anthocyanins are an essential component of dietary advice aimed at preventing metabolic disorders because of their ability to improve lipid metabolism, regulate blood glucose levels, and reduce inflammation (Chintha et al. 2023). Sweet potatoes are also rich in fiber, which enables individuals to maintain their weight, an essential factor in preventing obesity and its related diseases, such as T2D. High‐fiber foods allow individuals to have a healthy weight and reduce their chances of developing diabetes because they increase their feelings of fullness and lower their total calorie intake (De and De 2021).

A holistic approach to disease prevention could be enhanced by using sweet potatoes and other nutrient‐rich foods in daily diets. Because of their high nutrient content and potential therapeutic applications, sweet potatoes can be an essential component of a balanced diet designed to postpone the onset of chronic diseases, including diabetes, heart disease, and certain cancers (Jiang et al. 2020). Sweet potatoes are beneficial for patients with diabetes because of their high antioxidant profile, fiber content, and low glycemic index. Sweet potatoes can be easily added to one's diet and provide many benefits such as improved regulation of blood sugar, reduced inflammation, and enhanced antioxidant status. Sweet potatoes are an excellent addition to any diet that arranges health because they are a functional nourishment that can be significant to diabetes regulatory programs and assist with more general disease‐prevention measures. Both experimental and clinical studies have provided evidence of the functional benefits of sweet potatoes in terms of metabolic health and chronic disease prevention. In elderly patients with type 2 diabetes, a randomized controlled study of 54 patients showed that an enteral nutritional formula with white sweet potato significantly increased body weight, body mass index (BMI), Mini Nutritional Assessment (MNA) score, and Geriatric Nutritional Risk Index (GNRI), reduced HbA1c, and increased transferrin, high‐density lipoprotein (HDL) cholesterol, and vitamin A levels, without inducing untoward gastrointestinal symptoms (Chen et al. 2019). Similarly, in an open‐label 4‐week study of 20 overweight Caucasian adults, daily intake of a purple‐fleshed sweet potato beverage (250 mL, 234 mg anthocyanins) significantly reduced systolic blood pressure (*p* = 0.0125) without affecting safety parameters, and liver enzyme levels were normal (Oki et al. 2016). These findings are also supported by experimental research in a mouse model of high‐fat diet‐induced dry eye disease, in which supplementation with 5% purple sweet potato powder containing anthocyanins maintained lacrimal gland structure and function, decreased oxidative stress, and enhanced lipid homeostasis (Chiang et al. 2023). In food applications, the incorporation of sweet potato flour into baked products at a 20% replacement level of wheat flour has been shown to increase dietary fiber by 30% and antioxidant capacity without any impact on sensory quality (Tortoe et al. 2017). In addition, anthocyanin‐rich sweet potato extracts have also shown potent anti‐inflammatory activity in vivo and in vitro models, pointing toward their application in metabolic health interventions (Zhang et al. 2023). Together, these results support the merits of sweet potatoes as a functional food ingredient with a high nutritional value that can play a role in diabetes control and chronic disease prevention.

## 9 Conclusion and Future Perspectives

Sweet potatoes, *Ipomoea batatas* , which are expended globally, are a staple crop and, with their rich nutritional makeup and bioactive components, particularly anthocyanins, a hypothetically valuable functional food. Organic plant pigments, primarily present in several types of sweet potatoes, have been reported to possess potent anti‐inflammatory and antioxidant properties. Additional contributions of these nutrient‐dense food sources are the large portions they offer for dietary fiber, vitamins, minerals, and phytonutrients, increasing the health benefits of sweet potatoes. While focusing attention on anthocyanins obtained from sweet potatoes, which play an integral part in metabolic well‐being due to their reduced oxidative stress, enhanced insulin sensitivity, and mitigating inflammation effects, this review highlights how anthocyanins may potentially be applied in clinical therapy for the management and treatment of T2D as well as other diseases. This can be seen in the different modes of action of anthocyanins—scavenging free radicals, activation of antioxidant enzymes, and modulation of inflammatory pathways—which point out their significance in the diet for the defense of chronic diseases.

Future research should optimize anthocyanin bioavailability through advanced food‐processing and preservation technologies, such as encapsulation, to enhance stability and absorption. In addition, sweet potatoes can be included in personalized nutrition plans to fulfill specific health needs across various populations, especially in regions with highly dominant metabolic diseases. Advanced breeding and genetic modification may make these sweet potato types more nutrient‐rich and anthocyanin‐rich, and therefore more functional food items. However, sustainability and accessibility have the most significant impacts on the global health of sweet potatoes. Of course, malnutrition and chronic diseases can be abridged by hopeful manufacturing in areas where food insecurity occurs and by educating people about their culinary and health assistance. The significance of sweet potato anthocyanins in all‐inclusive dietary outlines and their interaction with other bioactive constituents is another area that should be investigated in future multidisciplinary studies.

## Author Contributions

**Sammra Maqsood:** writing – original draft (equal). **Nosiba S. Basher:** data curation (equal), formal analysis (equal). **Muhammad Tayyab Arshad:** writing – review and editing (equal). **Ali Ikram:** supervision (equal). **Douglas S. Kalman:** formal analysis (equal). **Md. Sakhawot Hossain:** data curation (equal), resources (equal). **Emmanuel Laryea:** validation (equal). **Nasir A. Ibrahim:** data curation (equal), visualization (equal).

## Disclosure

The authors have nothing to report.

## Ethics Statement

This study did not involve humans or animals.

## Consent

This study did not involve humans.

## Conflicts of Interest

The authors declare no conflicts of interest.

## Data Availability

The data supporting the findings of this study are available from the corresponding author upon reasonable request.
